# A nested bistable module within a negative feedback loop ensures different types of oscillations in signaling systems

**DOI:** 10.1038/s41598-022-27047-4

**Published:** 2023-01-11

**Authors:** Juan Ignacio Marrone, Jacques-Alexandre Sepulchre, Alejandra C. Ventura

**Affiliations:** 1 Departamento de Física, Facultad de Ciencias Exactas y Naturales, Universidad de Buenos Aires, Ciudad Universitaria, C1428EHA Buenos Aires, Argentina; 2 Instituto de Fisiología, Biología Molecular y Neurociencias (IFIBYNE UBA-CONICET), Consejo Nacional de Investigaciones Científicas y Técnicas of Argentina-Universidad de Buenos Aires, C1428EHA Buenos Aires, Argentina; 3 Institut de Physique de Nice, CNRS UMR7010, Université Côte d’Azur, 06200 Nice, France

**Keywords:** Systems biology, Biochemical networks, Dynamical systems, Multistability, Nonlinear dynamics, Oscillators, Signal processing

## Abstract

In this article, we consider a double phosphorylation cycle, a ubiquitous signaling component, having the ability to display bistability, a behavior strongly related to the existence of positive feedback loops. If this component is connected to other signaling elements, it very likely undergoes some sort of protein–protein interaction. In several cases, these interactions result in a non-explicit negative feedback effect, leading to interlinked positive and negative feedbacks. This combination was studied in the literature as a way to generate relaxation-type oscillations. Here, we show that the two feedbacks together ensure two types of oscillations, the relaxation-type ones and a smoother type of oscillations functioning in a very narrow range of frequencies, in such a way that outside that range, the amplitude of the oscillations is severely compromised. Even more, we show that the two feedbacks are essential for both oscillatory types to emerge, and it is their hierarchy what determines the type of oscillation at work. We used bifurcation analyses and amplitude vs. frequency curves to characterize and classify the oscillations. We also applied the same ideas to another simple model, with the goal of generalizing what we learned from signaling models. The results obtained display the wealth of oscillatory dynamics that exists in a system with a bistable module nested within a negative feedback loop, showing how to transition between different types of oscillations and other dynamical behaviors such as excitability. Our work provides a framework for the study of other oscillatory systems based on bistable modules, from simple two-component models to more complex examples like the MAPK cascade and experimental cases like cell cycle oscillators.

## Introduction

### Introduction to biochemical oscillators

Design principles of biochemical oscillators have been analyzed in detail^1–4^. Above all, the existence of a negative feedback loop is a necessary requirement for any nonlinear oscillator^5^. But it is not sufficient, since the negative feedback must be *delayed* in order to prevent the system from finding its homeostatic state by creating permanent overshoots and undershoots of the state variables in time^3^. There are several mechanisms to induce a delay in the negative feedback loop. One way is to incorporate an explicit delay in the modeling^6^. Another is to consider a reaction scheme with several intermediate steps, describing e.g., intermediate modifications or translocations of the biochemical species^2,7^. As discussed in Ref.^3^, an alternative way which mimics a delay is to submit the system to a *positive* feedback loop—the simplest instance being an auto-catalytic process—which can induce a hysteresis-type mechanism. Indeed, hysteresis is associated with internal memory of the system and naturally creates overshooting behaviors.

### Biological oscillators with two feedbacks

Biological oscillators having positive plus negative feedbacks have been studied and characterized in the literature^8^, addressing the question of what advantages the positive loop imparts, provided it is known that a simple negative feedback loop has the potential to generate sustained oscillations. While it is generally difficult to adjust a negative feedback oscillator’s frequency without compromising its amplitude, one can achieve a widely tunable frequency and near-constant amplitude by combining positive and negative feedback loops. Even more, interlinking both kinds of loops has been recognized as a fundamental ingredient to reveal a large repertoire of oscillatory types in nonlinear oscillators^8–19^. This class of systems can be embodied by a variety of 2-component models where one of the two species acts as a self-activator^2^. In this paper, we will analyze signaling motifs possessing more than 2 components but naturally characterized by both positive and negative feedback loops. As it will be mentioned, a crucial characteristic of these models is that without the positive feedback loop, no oscillations can be found. And, as it will be analyzed, when the positive loop is present, different types of oscillations arise.

From the literature, there are two main types of oscillators, classified according to the way they appear through bifurcations. The Type-1 oscillators are born through zero-frequency bifurcations such as the Saddle-Homoclinic (SHom) or Saddle-Node on an Invariant Circle (SNIC) bifurcation^20,21^. When the input parameters are varied around the bifurcation, the corresponding oscillations are characterized by relaxation oscillations, with a varying frequency going from zero to a maximum value, whereas the amplitude remains roughly constant. Generally, the amplitude-frequency diagram of Type-1 oscillations corresponds to a flat line^8^. The Type-2 oscillator comprises the finite-frequency oscillations, with a nearly harmonic profile, and characterized by Hopf bifurcations^20,21^. For supercritical Hopf bifurcations, when some input parameter is varied around the bifurcation, the amplitude of these oscillations varies between zero and a maximum value, while the frequency stays relatively constant within a small interval. The amplitude-frequency diagram of Type-2 oscillations is characterized by an “inverted-U” shape^8^.

### Single-stage bistability

Focusing on models of cell signaling pathways, the study by Qiao et al. analyzed the well-studied MAPK cascade model by Huang and Ferrell^22^, arriving at a large region of oscillatory responses^23^. They found that the emergence of oscillations requires at least two stages, where one of them should be based on double phosphorylation. From their results, the authors concluded that single-stage bistability is a necessary condition for the oscillatory behavior at the cascade level, which is consistent with double phosphorylation displaying bistability for particular choices of the parameter values^24^. They remarked that oscillations are built around a hysteresis loop, and that the oscillations in the MAPK cascade have relaxation character. In another study of the MAPK cascade, they focus on the substrate-dependent control of protein ERK, finding that the emergence of oscillations depends on bistability, similar to the canonical van der Pol oscillator^25^.

An article by Suwanmajo and Krishnan studied the behavior of multisite phosphorylation systems as part of signaling pathways^26^. They presented a minimal oscillator based on a double phosphorylation cycle, where oscillations were induced by the presence of linear enzyme activation/inactivation. This model is of particular interest since removing the variables corresponding to the inactive enzymes recovers the double phosphorylation cycle, which is incapable of oscillations on its own.

### Importance

Previous works in systems biology have analyzed different types of oscillations for different system designs^1,8,27^. In the current paper, we show that interlinked positive and negative feedbacks, leading to a nested bistable module within a negative feedback loop, can produce different types of oscillations ranging from Type-1 to Type-2 within the same system design. We focus on two models with double phosphorylation, of several components each (higher than 2), and we study them without reducing the number of variables. As mentioned, bistability in the double phosphorylation cycle is a necessary condition for oscillations to occur, meaning that a positive feedback loop is present. Our analysis puts focus on whether the positive or the negative loop is the one dominating the oscillations in the system, and how the output relates to the underlying bistability. Bifurcation analysis, amplitude vs. frequency curves, and time series provided the information necessary for our studies.

Understanding how oscillations arise in signaling systems is an important and well-studied subject. However, the mechanism we are proposing here is novel in its role of producing two different types of oscillations. It relies on bistability and sequestration. Sequestration is produced by binding, so it takes place everywhere in signaling pathways. Its magnitude and, thus, its relevance and effects depend on the system’s parameter values. Bistability can be achieved in different signaling structures, being double phosphorylation one of them. Enzyme sharing was shown to lead to bistability^28^, and given the ubiquity of this process, it is likely that bistability is a frequent feature in signaling. Summarizing, the ingredients we are considering are likely to appear together in most signaling pathways. For this reason, it is important to understand the biological functions that can emerge from them, particularly two different types of oscillatory behavior and the possibility of tuning the system from one type to the other, with clearly different properties, or somewhere in between, with mixed properties.

### Organization of the paper

The organization of the paper is as follows. We first study a double phosphorylation cycle with minimal additions that ensure sequestration. We identify two types of oscillatory behavior, and a path in the parameter space connecting both of them which evidences the change in feedback hierarchies. We then apply a similar study to the first part of the MAPK cascade, finding similar results. After that, we evaluate the performance of a simplified model that deals with a different bistable module. Finally, we analyze an oscillatory model based on experimental data in its ability to coexist, in a different and non-explored region of the parameter space, with another type of oscillations. We then conclude by discussing the potential implications of the results in the article. All parameter values are provided in Methods and in the Figures.

## Results

### A minimal signaling model produces a variety of oscillations

**Figure 1:**
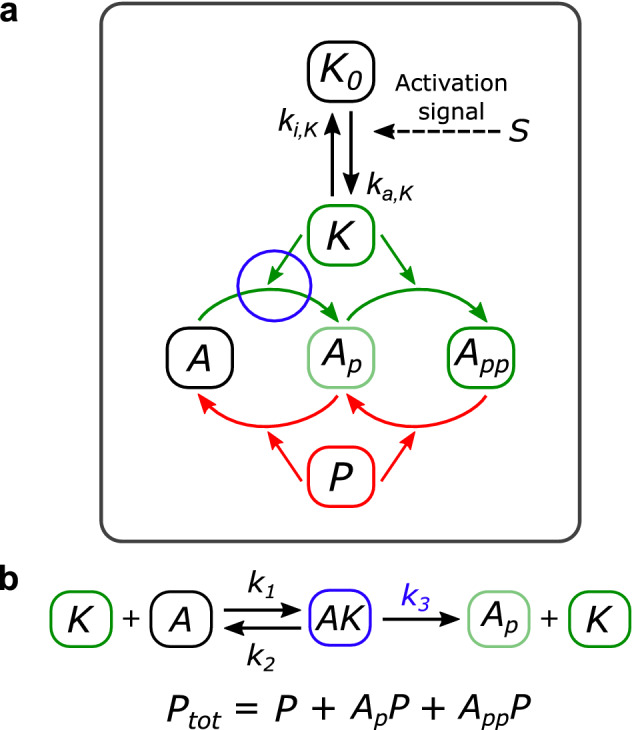
Scheme of the Suwanmajo-Krishnan model, based on their Figure^26^. (**a**) In green, the phosphorylation reactions. In red, the dephosphorylation ones. Each of these reactions involves the association, dissociation, and catalysis of an intermediate complex. The activation and inactivation of the first-level kinase is direct (no intermediate complex). The input is the parameter S, the output A_pp_. (**b**) Shown for later results: the reactions in the first phosphorylation step and the variables making up the total amount of phosphatase.

We focus on a model proposed by Suwanmajo and Krishnan^26^, consisting of a double phosphorylation cycle (DP cycle) presenting one shared kinase and one shared phosphatase, with the addition of an activation module for the kinase (Fig. 1a, see Supplementary Fig. S1 for a scheme of the DP reactions). This module, where the activation and inactivation are both direct, allows for an extra variable with respect to the isolated DP cycle, the inactive kinase, and can represent the connection between the previously isolated module and a signaling pathway. Thus, the resulting model combines a structure capable of displaying bistability, possible due to underlying positive feedbacks^29,30^, with the sequestration of an enzyme, in turn producing a non-explicit negative feedback^31^. By non-explicit feedback we mean a feedback structure which is not explicitly apparent on the biochemical network. For example, Fig. 1a does not explicitly convey the presence of a negative feedback loop which will be discussed later. The relative simplicity of the system, which only adds one variable to a DP cycle, together with the combination of positive and non-explicit negative feedbacks, makes this model of particular interest in the study of oscillations in signaling systems. In this work, we aim to analyze how to obtain different types of oscillations by manipulating the timescale separation between the inactive kinase and the DP cycle, the sequestration of the free active kinase, and the bistable behavior of the DP cycle.

#### Different oscillatory outputs by modifying the timescale separation with the inactivation constant

Starting from the ODE model from Suwanmajo and Krishnan^26^, it is possible to rewrite the equation corresponding to the inactive kinase, such that the inactivation rate constant multiplies the rest of the equation. From our computational studies, we decided to take k_a,K_ = k_i,K_, since for this condition the system exhibits oscillatory behavior. This way, the inactivation constant acts as a timescale separation parameter between the inactive kinase and the rest of the variables (see “Methods” section for the ODE system and the rewritten equation).

**Figure 2:**
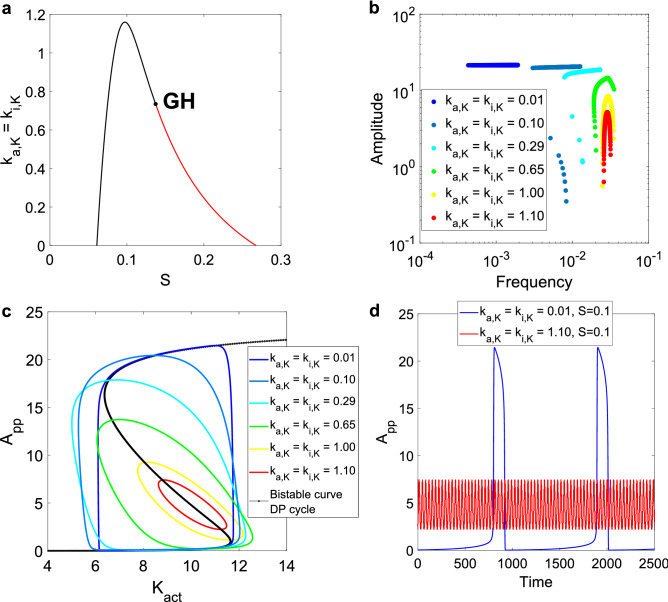
Results for the timescale separation analysis of the SK model. (**a**) 2D bifurcation diagram of k_i,K_ and S, with k_i,K_ = k_a,K_. In black, the supercritical Hopf curve. In red, the subcritical curve. The Generalized Hopf (GH) bifurcation is included in black. The oscillatory region is bordered by the x-axis and both Hopf curves. The S range where oscillations are found increases with the timescale separation. (**b**) Amplitude vs. frequency curves for six different values of k_i,K_ = k_a,K_, scanning the input parameter S. As the timescale separation increases, the curves move up and left, going from wide amplitude ranges and small frequency ones to the inverse case. For the lowest values of k_i,K_, some low amplitude values can be found on the left branch of the curves, where there is a supercritical Hopf, if the parameter is precise enough. (**c**) One limit cycle for each of the six previous cases (with S = 0.1), plotted against the underlying bistable curve of the DP cycle, while scanning the active kinase parameter (K_act_). The limit cycles take more advantage of the bistable amplitude as k_i,K_ decreases. (**d**) Time series for the two extreme cases, showing the clear difference in amplitude and frequency.

In Fig. 2, we show the results derived from studying the system with different timescale separation values. In Fig. 2a, we present a 2D bifurcation diagram for k_i,K_ vs. S, the signal input (as mentioned, k_i,K_ is equal to k_a,K_). Each point of the curve represents a Hopf bifurcation, and each parameter set below this curve corresponds to an oscillatory point. A Generalized Hopf (GH) bifurcation is found, separating supercritical (in black in Fig. 2a) from subcritical bifurcations (in red). This defines two regions: above the GH, a scan in S results in two supercritical Hopf bifurcations, while below the GH, a supercritical Hopf occurs to the left of the oscillatory region and a subcritical Hopf to the right. This diagram allows to choose a given value of k_i,K_, change the activation signal S, and find different Hopf bifurcations.

In Fig. 2b, we present amplitude vs. frequency curves for six selected values. These curves are obtained from scanning S, and correspond to the oscillatory characteristics of the output variable, i.e., the double-phosphorylated substrate A_pp_. According to a previously reported classification of oscillators, those based on only negative-feedback exhibit an amplitude vs. frequency curve with the form of an “inverted U”, while those arising from positive-plus-negative feedback present mostly flat curves^8^. From the curves obtained for different k_a,K_ and k_i,K_ values, it is evident that both the width of the amplitude and frequency ranges, and the raw values of amplitude and frequency, are strongly dependent on the timescale separation. For k_a,K_ = k_i,K_ = 1, the curve has an “inverted U” shape, displaying a wide range of amplitude values as opposed to a small range for the frequency. This is consistent with the results for the negative-feedback models studied in Ref.^8^, even though the model analyzed in our work presents both positive and negative feedbacks.

As the timescale separation is increased (lower values for k_a,K_ = k_i,K_), the amplitude vs. frequency curve changes its shape, allowing a wider range of frequencies but a smaller range for the amplitude. The positive-plus-negative feedback oscillators from Tsai et al. display these amplitude-robust and frequency-sensitive behavior^8^. The authors also present a cell cycle model for which they can adjust the curve, by changing a parameter responsible for the strength of the positive feedback. In our studied model, changing the values for k_a,K_ = k_i,K_ does not affect the positive feedback, but it can be interpreted as changing the relative strength of the positive and negative feedback loops. Indeed, the inactive kinase can be seen as a sequestrating variable, and as it will be explained below with more details, the activation module plays the role of a negative feedback loop for the DP module. Therefore, when the values of the activation module constants are decreased, the strength of the negative feedback is also decreased when compared with the positive loop.

In terms of the amplitude and frequency values, it is shown how a larger timescale separation increases the former and decreases the latter. A change in the amplitude and frequency of the oscillations is studied in the previously mentioned cell cycle model in Ref.^14^. The authors point out that the system effectively acts as a negative-feedback oscillator for the lower amplitude and higher frequency case. While our work deals with a minimal signaling model with non-explicit feedbacks, the results obtained in terms of the timescale separation show the possibility of transitioning between different modes of oscillations.

When k_a,K_ and k_i,K_ are decreased, the bistable module (the DP cycle) has faster reactions compared to those in the kinase activation module. In this case, the bistable dynamics are faster than the sequestration occurring in the first level of the model. In Fig. 2c, we present a comparison of limit cycles obtained in the Suwanmajo–Krishnan model and the corresponding bistable curve of the isolated DP cycle. The total amount of active kinase, K_act_, is the sum of the free active kinase plus the two intermediate complexes in each phosphorylation step. K_act_ goes from being a parameter in the DP cycle to a variable in the extended system and allows us to evaluate the relationship between the subsystem’s bistability and the emergent oscillations. Since the only parameters that are being modified correspond to the activation module, the underlying bistable curve remains the same. When the timescale separation is large, the limit cycle closely follows the hysteresis cycle that arises in the bistable module, showing a typical relaxation oscillation. As the timescale separation diminishes, the limit cycle becomes both smoother and smaller, while still being determined by the position of the bistable curve. In Fig. 2d, we show the time series for the two extremes, and how they differ in amplitude and frequency. This is further evidence that the simple activation module can switch the system between different oscillatory modes.

#### New bifurcations by manipulating the underlying bistability

A certain degree of timescale separation is typically found in relaxation oscillators, and as we have shown in the previous subsection, increasing it allows for higher values of amplitude and period, together with a spike-type shape. However, it is possible to obtain different types of oscillations by fixing the timescale separation, and instead, manipulate the bistability found within the system. We proceed to study what happens in the DP cycle when modifying a specific parameter, and how the behavior of the bistable module translates to the oscillations in the full system.

**Figure 3:**
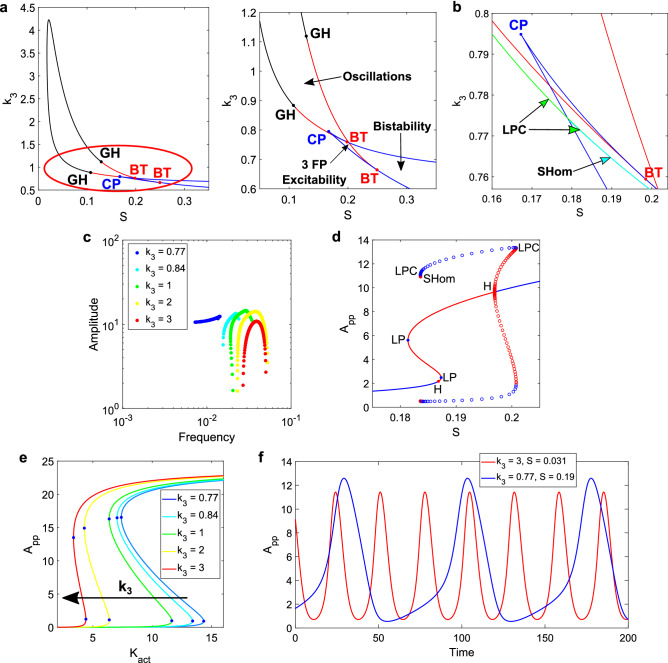
Results for the k_3_ vs S analysis of the SK model. (**a**) 2D bifurcation diagram for k_3_ and S. In black, the supercritical Hopf curve. In red, the subcritical one. In blue, the Saddle-Node (SN) curves, with the Cusp Point (CP) at the joint, also in blue. The GH bifurcations in black. The Bogdanov-Takens (BT) bifurcations are in red. A bistable region develops for low values of k_3_ and high values of S, separated from the excitable region by a portion of the subcritical Hopf curve. (**b**) Zoom of the 2D bifurcation diagram with the Saddle-Homoclinic (SHom, in cyan) and Limit Point Cycle (LPC, in green) curves. The stable limit cycles end at the LPC curves, but the unstable ones increase their period as they approach the SHom bifurcations. (**c**) Amplitude vs. frequency curves for five different values of k_3_. Inverted “U” near the top of the oscillatory region, and a transition in the shape as the system approaches the bistable and excitable regions. More robust amplitude, lower frequency values and relatively larger ranges are observed for lower k_3_. (**d**) 1D bifurcation diagram for k_3_ = 0.77 and a scan of S. Steady states: stable in blue lines, unstable in red. Same colors for the limit cycles and circles. An unstable limit cycle is born from the right-hand side subcritical Hopf and continues until the LPC. The stable limit cycle is limited by the LPCs. An unstable limit cycle is limited between the left LPC and an SHom (in a very small range of S). The left subcritical Hopf is “disconnected” from the left LPC: starting from said Hopf, an unstable limit cycle runs for a small range of S and ends at another SHom branch (not shown). (**e**) Bistable curves in the DP cycle while scanning K_act_, for each of the five cases studied. SN points in blue. Clear change in the range for the active kinase, allowing changes in the oscillator frequencies, while the amplitude varies slightly for each k_3_ value. (**f**) Time series for two pairs of k_3_ and S, representing the variety of oscillatory outputs.

In Fig. 3, we show the same type of analysis presented in Fig. 2, this time studying the effect of the catalytic rate constant for the first phosphorylation step: the parameter k_3_ (see Fig. 1b for the scheme of the reaction). For all these results, k_a,K_ = k_i,K_ = 0.65, which represents a relatively small degree of timescale separation.

In Fig. 3a, the 2D bifurcation diagram for k_3_ vs. S shows the appearance of not only GH bifurcations, but also Bogdanov–Takens (BT) bifurcations and a Cusp Point (CP). This is relevant to our studies, since the presence of BT and CP is evidence of Saddle-Homoclinic (SHom) and Saddle-Node (SN) bifurcations in the system^32^. An SHom bifurcation has a drastic effect on the oscillations: near this bifurcation, the amplitude remains constant while the period increases significantly. An SN bifurcation indicates that there is bistability in some region of the parameter space. It is possible to define three regions accordingly in this 2D diagram: one oscillatory, one excitable (of three fixed points) and one bistable (as indicated with arrows in the enhanced version of Fig. 3a).

In Fig. 3b, we show bifurcation curves not included in the previous panel: SHom and Limit Point Cycle (LPC). These curves are very close in distance. The presence of a subcritical Hopf curve is consistent with an LPC curve since the limit cycles born from said Hopf are unstable up to a bifurcation (the LPC), where they collide with a stable limit cycle. The Saddle-Homoclinic curve appears between the SN curves, where a saddle point can be found.

For high values of k_3_ within the oscillatory region, the bifurcations correspond to supercritical Hopf’s, consistent with the amplitude vs. frequency curves presented in Fig. 3c, for k_3_ = 2 and k_3_ = 3, and varying S. The frequency range is small relative to the amplitude range: the amplitude is easier to manipulate. For lower values of k_3_, the system approaches the SHom bifurcations and the bistable region, resulting in smaller amplitude ranges. At k_3_ = 1, the right-handed Hopf bifurcation is now subcritical, explaining the limited values for the amplitude seen on this right branch of the curve. For k_3_ = 0.84, both ends come from subcritical Hopf’s, narrowing the total amplitude range. On the other hand, the frequency values move to the left as k_3_ is lowered.

At k_3_ = 0.77, an SHom bifurcation arises to the left of the oscillatory range for S, and the amplitude becomes quite robust. Even though this bifurcation is present, the stable limit cycle ends at the LPC before the period can reach higher values. This is reflected in the somewhat limited frequency range observed in the blue curve of Fig. 3c (to note that the axis is in log scale).

In Fig. 3d, we show the 1D bifurcation diagram for k_3_ = 0.77 while scanning the input S. The SHom bifurcation is connected to an unstable limit cycle, which sees its period grow to increasingly higher values. This prevents the system from taking advantage, through its stable limit cycles, of the period increase (see Figure legend for a description of the curves).

The behavior of the amplitude vs. frequency curves is closely related to the underlying bistability of the DP cycle. In Fig. 3e, the bistable curves for the k_3_ values studied in the full system are shown. As k_3_ decreases, the bistable range becomes wider, explaining the longer periods found: the wider the range, the more time it takes for the system to complete one cycle. On the other hand, the amplitude remains nearly identical. A stronger bistable behavior in the DP subsystem leads to different modes of oscillation in the complete system, to the point where it is now possible to find bistability. For a sufficiently low value of k_3_, the DP cycle + kinase activation model displays bistable behavior, in a “transfer of bistability”^33^ from the subsystem towards the signaling motif that includes the activation step. This means that the bistability in the DP cycle becomes strong enough that now the full system is bistable, while the oscillations occurring in the vicinity of the bistable region are significantly different from the ones found at the opposite extreme of the k_3_ oscillatory range.

At this point, it is important to make two remarks. First, these results did not arise when controlling just the timescale separation. In that case, only a Generalized Hopf bifurcation was found. There was no “transfer of bistability”, since the DP cycle remained the same when modifying k_a,K_ and k_i,K_. Second, only one parameter was changed in this subsection: k_3_. By selecting a parameter in the DP cycle capable of changing the characteristics of the underlying bistability, the oscillations in the full system can be controlled.

In Fig. 3f, two examples of oscillations are shown, for opposite ends of the k_3_ oscillatory range. There is an evident change in the period, while the amplitude is similar, as was shown in the different amplitude vs. frequency curves. For k_3_ = 0.77, it is possible to change the value of S and obtain different shapes for the oscillations (see Supplementary Fig. S2).

#### Control of amplitude and frequency with two DP cycle parameters

**Figure 4:**
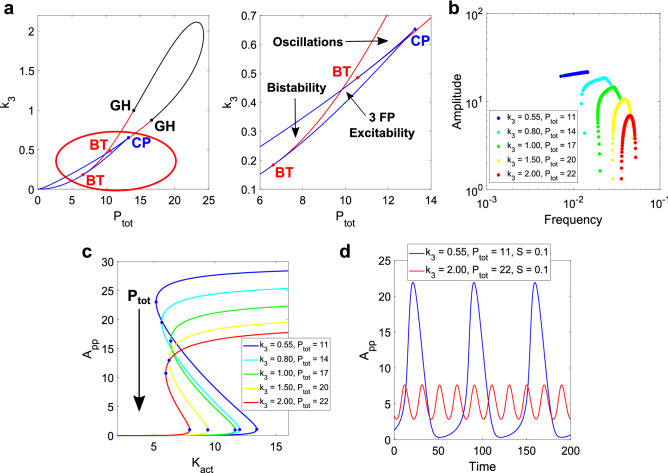
Results for the k_3_ vs P_tot_ analysis in the SK model. (**a**) 2D bifurcation diagram for the two parameters. In black, the supercritical Hopf curve. In red, the subcritical one. In blue, the SN curves with the CP at the joint. GH bifurcations in black. BT bifurcations in red. A bistable region develops for low values of k_3_ and low values of P_tot_. (**b**) Amplitude vs. frequency curves for different pairs of k_3_ and P_tot_, scanning the parameter S. A transition in the shape of these curves is observed like in the previous analysis, the amplitude now reaching higher values than when only modifying k_3_. (**c**) DP bistable curves while scanning K_act_ for each pair selected, showing the clear increase in amplitude as P_tot_ is lowered. (**d**) Time series for two extreme cases, showing the contrast in shapes within the oscillatory region.

It is possible to take advantage of the variety of parameters available in the DP cycle to further manipulate the bistability and analyze its impact on the oscillations. In Fig. 4, we present the results obtained from varying two DP parameters: k_3_ and P_tot_, the latter being the total amount of phosphatase (see Fig. 1b for the variables making up this conserved amount). While k_3_ may represent a difficult-to-change reaction, it would be doable to modify the total amount of phosphatase from an experimental point of view (although a change in phosphatase level may affect other pathways).

In Fig. 4a, we show a 2D parameter scan in the plane k_3_ − P_tot_. This required a fixed value of S. For this, we chose S = 0.1, at which the range of k_i,K_ displaying oscillatory behavior was near its widest (see Fig. 2a).

Like for k_3_ vs. S, the variation of the two chosen DP parameters shows an oscillatory region that is next to a bistable region, and an excitable one. Once again, BT bifurcations appear, with SHom curves present in the vicinity.

We then chose different pairs (P_tot_, k_3_) as to cover the oscillatory region and performed a parameter scan of S for each pair. In Fig. 4b, we present the amplitude vs. frequency curves for each pair chosen. At high values of k_3_ and P_tot_, the curve forms an inverted “U” with a small frequency range compared to the amplitude one. As the pair (P_tot_, k_3_) approaches the bistable region, the frequency range widens. At the same time, the amplitude values increase, while the frequencies decrease. Once again, this is related to the underlying bistable behavior in the DP cycle. In Fig. 4c, the DP curves for each pair selected are shown. Unlike the k_3_ case, here the amplitude shows a significant change. Increasing the amount of phosphatase results in lower amplitude for the double-phosphorylated output, which is consistent with its role. At the same time, since k_3_ is also different for each curve, the K_act_ range changes. Both parameters then explain why the amplitude and frequency values for the oscillations depend on the pair chosen. Take, for example, the case k_3_ = 2 and P_tot_ = 22 (red curves in Fig. 4b,c). If the bistable amplitude is lower, then the oscillatory amplitude values will be consequently lower. And if the bistable range is narrower, the period values will be consequently shorter, so the frequency will be higher.

In Fig. 4d, we show a couple of examples of oscillatory time series, for the two extreme cases selected. Even though both take place at same value for k_a,K_ = k_i,K_, it is clear the difference in amplitude and frequency between them. These results indicate that controlling the underlying bistability in an oscillator can lead to highly different outputs.

As we showed for parameter k_3_, it is also possible to obtain a “transfer of bistability”^33^ while appropriately lowering k_3_ and P_tot_. For a sufficiently high degree of bistability in the DP cycle, the system will display amplitude values that are both high and robust to changes in the input. But eventually, the underlying bistability muzzles the effect of the negative feedback. The oscillations will disappear, and now the full system will present bistable behavior.

#### The catalytic rate k_3_ regulates the hierarchy of feedbacks

As was shown in the previous subsections, controlling the parameters of the DP cycle has an important effect on the oscillations displayed by the full system. In particular, the catalytic constant for the first phosphorylation step, k_3_, is able to shape the width of the underlying bistable curve. And for a sufficiently low value, a “transfer of bistability” occurs: not only the isolated DP cycle is bistable, the full system now also displays this behavior. On the other hand, high values of k_3_ result in amplitude vs. frequency curves and bifurcations expected in a Type-2 (or Hopf-type) oscillator.

The k_3_ parameter plays a key role in how the free active kinase (K) reacts with both modules, the kinase activation one and the DP cycle. It controls part of the second-level sequestration of K. That is, the binding of the second-level unphosphorylated substrate (A) to K, to form an intermediate complex (AK, see Fig. 1b). This complex protects K from the first-level sequestration, which comes in the form of a direct inactivation.

The non-explicit negative feedback in this system is a result from the first-level sequestration, which competes with substrate A to react with K. A higher value of k_3_ means that the protection against kinase inactivation given by AK is weakened, since this complex reacts faster to give the product, consisting of the single-phosphorylated substrate (A_p_) and K. With the active kinase once again free, the first level can sequester it for deactivation and therefore decrease A_pp_. This means that raising k_3_ can increase the effect of the negative feedback over the positive one, present in the double phosphorylation cycle. This gives the advantage to the negative feedback in the hierarchy.

In the opposite case, lowering k_3_ increases the protection against inactivation given by the bound state AK, thereby turning down the effect of the negative feedback. This explains why both in the k_3_ vs. P_tot_ and the k_3_ vs. S bifurcation diagrams, high values of k_3_ result in a pair of supercritical Hopf bifurcations when scanning the input parameters, while low values of k_3_ bring the system to both subcritical Hopf and Homoclinic bifurcations. For even lower values of k_3_, the system visits a bistable region, where the positive feedback completely silences the presence of the negative feedback.

In summary, not only the variation of k_3_ regulates the strength of the positive feedback in the DP cycle (by regulating the width of the bistable curve), it also modulates the combined effect of the interlinked positive and negative feedbacks, the latter being born from the first-level sequestration of the free active kinase.

### The two-level MAPK cascade enriches the oscillatory behavior with respect to the minimal model

In the previous section, we analyzed an oscillator based on the bistability of a DP cycle, which only adds one variable (the inactive kinase) to the bistable module. This model, which we will call “SK” following the authors’ initials, appears to be the simplest oscillator that can be obtained when combining a DP cycle with the sequestration of one of its enzymes (in Suwanmajo and Krishnan’s paper, they also present the case of adding inactive phosphatase instead of inactive kinase^26^). In this section, we study the MAPK cascade truncated to its two first levels. This signaling motif is a natural extension of the previous model and gets closer in its description to the full Huang-Ferrell cascade, one of the most studied systems to describe signaling pathways^22^.

**Figure 5:**
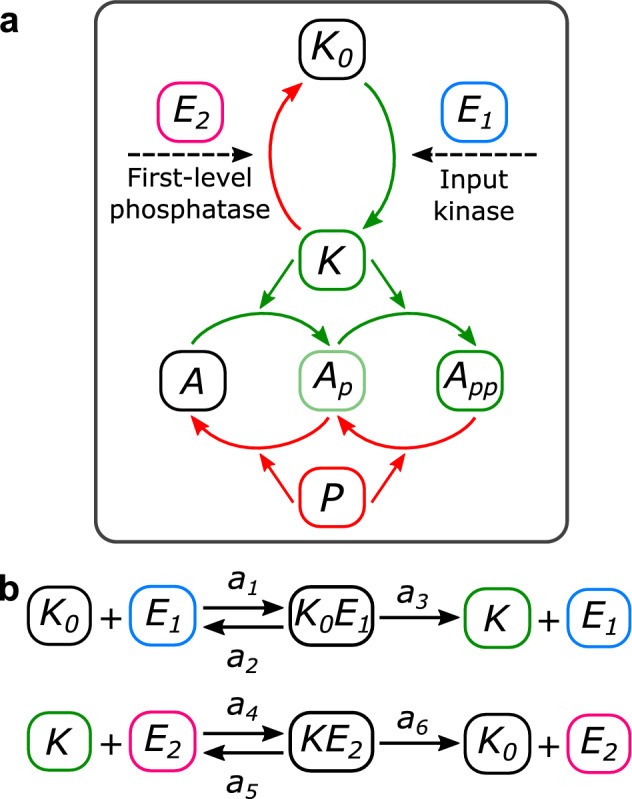
Scheme of the 1 + 2 system and its enzymatic first-level reactions. (**a**) Scheme of the model, with the kinase E_1_ and phosphatase E_2_ involved in the first-level reactions standing in contrast to the SK activation module. (**b**) Reactions for the activation and inactivation of the first-level kinase, involving intermediate complexes with E_1_ and E_2_.

In Fig. 5a, we show the scheme for the two-level MAPK cascade, where the activation and inactivation of the kinase in the first level occur through association, dissociation, and catalysis. These enzymatic reactions, shown in Fig. 5b, stand in contrast to the direct activation and inactivation taking place in the SK model. Four additional variables are needed (input kinase E_1_, phosphatase E_2_, and the two intermediate complexes, see Fig. 5b), and six rate constants are involved (when only two appeared in the SK motif). The input parameter is the total kinase E_1tot_ (conserved amount distributed between E_1_ and the corresponding intermediate complex K_0_E_1_).

This system has been studied by Qiao et al., in a paper where the full Huang-Ferrell cascade is analyzed^23^. Since it corresponds to the first two levels of said cascade, they call it the “1 + 2” model. This is how we will refer to it from now on. (It can also be interpreted as a 1 + 2 model since it combines a single phosphorylation cycle with a double one). We aim to show how to obtain different types of oscillations in this model, and how the method is quite like the one applied for the minimal oscillator considered in the previous section (see “Methods” section for the system of equations).

**Figure 6:**
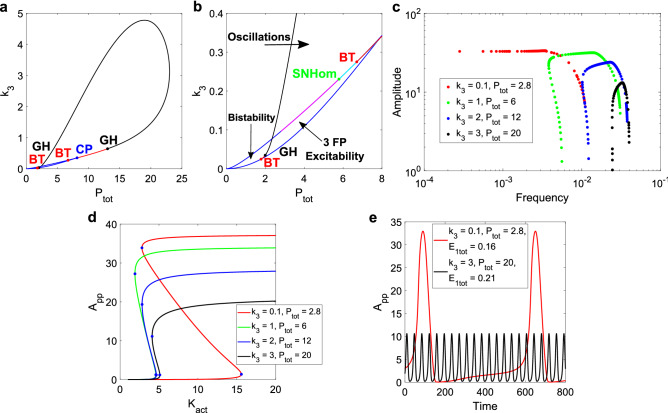
Results for the k_3_ vs P_tot_ analysis in the 1 + 2 model. (**a**) 2D bifurcation diagram for the two parameters. In black, the supercritical Hopf curve. In red, the subcritical one. In blue, the SN curves with the CP at the joint. GH bifurcations in black. BT bifurcations in red. The oscillatory region is considerably larger than for the SK model. (**b**) Zoom of the 2D diagram, showing the different regions and the global bifurcations. Like in the SK model, a bistable region develops for low values of k_3_ and low values of P_tot_. The Saddle-Node on Invariant Circle (SNIC) curve is in magenta, following the path of one of the SN curves. This SNIC curve connects to a relatively small SHom curve via a SNHom point. Then the SHom curve (in cyan) connects to the Hopf curve via the BT bifurcation. (**c**) Amplitude vs. frequency curves for four different pairs of k_3_ and P_tot_, scanning the parameter E_1tot_. There is more flexibility in the transition of these curves when compared to the SK model. A SNIC bifurcation allows the frequency to be significantly lowered for the pair closest to the bistable region. Supercritical Hopf bifurcations allow the amplitude to reach lower values in the other branches of the curves, although the scan of the input becomes more precise close to the bifurcations as the system moves to the lower left of the 2D diagram. (**d**) DP bistable curves while scanning K_act_ for each pair selected, showing, as before, the increase in amplitude with lower P_tot_. (**e**) Time series for two extreme cases within the oscillatory region.

In Fig. 6, we present the results for the analysis in the k_3_ − P_tot_ plane, as we did in the SK model. The values are the same for all the parameters that the 1 + 2 and SK system have in common (every rate constant in the DP cycle, the total amount of MAP kinase in the first level, substrate in the second level, and phosphatase for the second level). All rate constants in the first level are equal to 1, and by scanning the input E_1tot_, an oscillatory range emerged.

The shape of the Hopf curve in Fig. 6a is comparable to the one found for the SK model. This is consistent, since the parameters being scanned belong to the same DP module. Once again, a bistable region develops for low values of k_3_ and P_tot_, along with a 3FP excitable zone. In Fig. 6b, we indicate these regions and the global bifurcations found, which are both Saddle-Homoclinic (SHom) and Saddle-Node Homoclinic on an Invariant Circle (SNIC).

These global bifurcations are responsible for delineating the oscillatory region once the Hopf curve ends at the BT point. It is possible that for the SK model, SNIC bifurcations could appear for other combinations of the system parameters. However, it is interesting that given the same values for all the shared parameters, this other global bifurcation emerges.

Furthermore, when comparing the two systems, one can easily see that the oscillatory region in the k_3_ – P_tot_ plane is larger in the 1 + 2 case. This is mainly due to the extended oscillatory range for k_3_. As we said, the parameter values in the DP cycle have not changed from the SK model, meaning that the bistable region in the isolated DP cycle remains the same. In particular, the range for k_3_ where it is possible to find bistable behavior in that module has not been changed (see Supplementary Fig. S3). This means that the wider oscillatory range comes from the different structure of the first level.

In the Supplementary Information, we show how the oscillatory region for k_3_ vs. S expands in the SK model as the timescale separation increases (Supplementary Fig. S4). In this section, we are showing a similar effect by changing the mechanism that activates the kinase, now involving association, dissociation, and catalysis reactions. The system takes more advantage of the underlying bistability for the development of oscillations.

Moreover, while the oscillatory region found for the 1 + 2 model is larger than the one obtained for the SK system, most of the increase comes from the region limited by the supercritical part of the Hopf curve. The two GH bifurcations stand below k_3_ = 1, with one of them standing in the boundary of the oscillatory region (the other is in the segment of the curve that separates the 3FP excitable region from the bistable one). For the SK sets of parameters, the GH with the highest k_3_ value stood at k_3_ = 1. So, in both cases, the Hopf curve above k_3_ = 1 is made up of supercritical bifurcations. Since the extended oscillatory range for k_3_ in the 1 + 2 case is based on this longer supercritical portion of the Hopf curve, there is a wider region for finding oscillations of tunable amplitude and robust frequency.

In Fig. 6c, we show amplitude vs. frequency curves for four different pairs (P_tot_, k_3_) within the oscillatory region. As we did for the SK model, we scanned the input parameter for each case, finding similar results: a wider range of frequencies, lower frequency values, and a higher and more robust amplitude as the system moves to lower values of k_3_ and P_tot_.

At the same time, the transition from a curve with robust amplitude to an inverted “U” has some noticeable differences compared to the results for the SK model. It is possible to find wider ranges of frequencies while still significantly lowering the amplitudes at the extremes of the oscillatory range. Comparing with the curves found in the SK model, it is evident that the intermediate curves display more variation in the frequency. The fall in the amplitude values is due to the prevalence of supercritical Hopf bifurcations, which limit all eight ends of the four curves, except for the k_3_ = 0.1, P_tot_ = 2.8 case. This last set has a SNIC bifurcation, allowing for a substantial decrease of the frequency while leaving the amplitude fixed. Compared to the robust amplitude curve obtained for the SK model, it is clear the difference in the frequency range. There is no LPC curve ending the stable limit cycle, like it was the case for the previous model. The expansion in the oscillatory region that occurs in the 1 + 2 case while changing k_3_ and P_tot_ leads to amplitude vs. frequency curves capable of more flexibility in their shape with respect to the previous model.

Each of the amplitude vs. frequency curves has its corresponding bistable curve in the DP cycle, shown in Fig. 6d. The relationship between them is like the one found in the SK system: lowering k_3_ increases the bistable range, and a decrease in P_tot_ leads to an increase in the amplitude, resulting in lower frequencies and higher amplitudes in the oscillator. Finally, time courses are presented in Fig. 6e.

These results for the 1 + 2 analysis show that the simplification of the reactions in the first level, that took place in the SK model, is not a requirement to obtain different types of oscillations. Direct activation or inactivation of the kinase simplifies the analysis, especially when changing the timescale separation between the levels of the cascade. Even then, reducing by an order of magnitude all six rate constants in the 1 + 2 first level shows similar results to the Krishnan model (see Supplementary Fig. S5).

The added complexity of the 1 + 2 model keeps the different characteristics of the Suwanmajo-Krishnan model, while enhancing the oscillatory region and allowing lower frequencies due to the SNIC bifurcation.

### The activator-inhibitor model resumes the different types of oscillations with only two components

In this section, we analyze a 2-component model with bistability arising from autocatalysis: an activator-inhibitor model. This has two purposes: one, to show an example that has only two variables, contrary to the SK and 1 + 2 models that involve a relatively large number of variables and parameters; two, to present a case where the bistability doesn’t come from a DP cycle, and yet the results can be obtained as before.

Previous works deal with variations of the activator-inhibitor model. Sensse and Eiswirth mention how it is possible to find 2D bifurcation diagrams involving Hopf, Saddle-Nodes, BT, and CP bifurcations^34^. Krishna et al. study the emergence of oscillations from the underlying bistability, making an analogy with the ferromagnetic model of spins by describing the oscillations as a case of frustrated bistability^35^. Here, we show how to take advantage of the bifurcations and underlying bistability to find different behaviors in the oscillations, as we have done for the DP cycle models.

**Figure 7:**
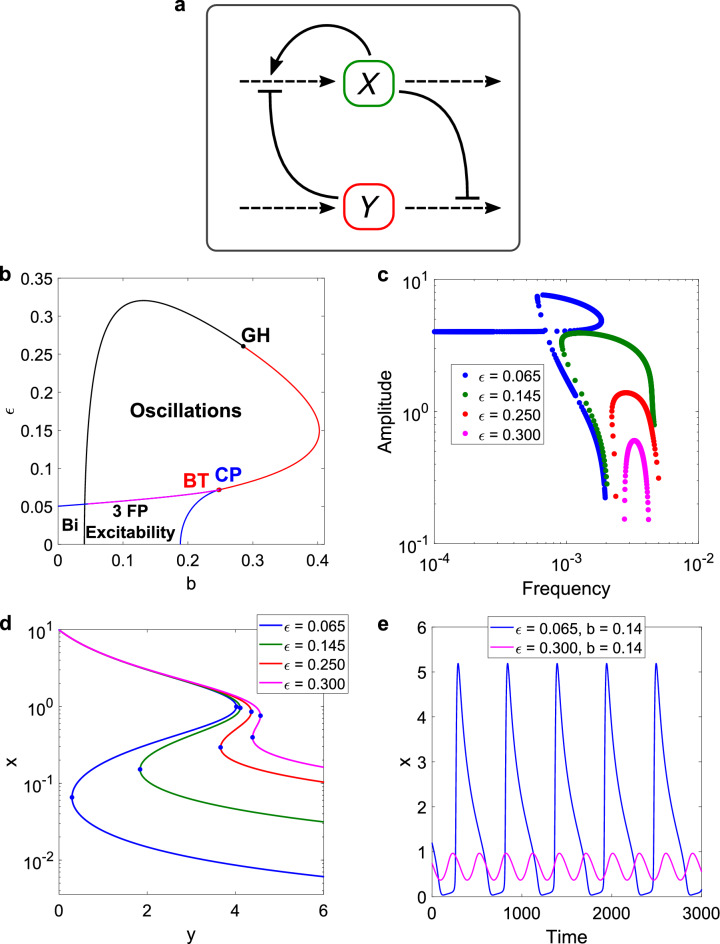
Scheme and results for the activator-inhibitor model studied by Tyson, based on his work^2^. (**a**) Scheme: both the activator x and inhibitor y go through production and degradation reactions. The activator has an auto-catalytic loop, while forming a negative feedback loop with the inhibitor. x inhibits the degradation of y, and in turn y inhibits the production of x. The input considered will be the production of the inhibitor (parameter b), and the output is the activator level. (**b**) 2D bifurcation diagram for the scanning of parameters ε and b. In black, the supercritical Hopf curve. In red, the subcritical one. In blue, the SN curves with the CP at the joint. GH bifurcation in black. BT bifurcation in red. The BT and CP bifurcations are close to each other. In magenta, the SNIC curve (contained within the SN curve). This curve joins an SHom curve via a SNHom (not shown due to the very small size of the SHom). For low values of ε, bistable and excitable regions emerge. Low values of the input are needed to reach the bistable region. (**c**) Amplitude vs. frequency curves in four different cases for ε when scanning the input parameter b. Transition in the shape of the curves as ε is changed, with a loop curve for the lowest value. A SNIC is responsible for the lowering of the frequency in this extreme case for ε. For this, and for the lowering of the amplitude at the other end of the curve, the scan of the input parameter is very precise. (**d**) Bistable curves for the auto-catalytic variable, for each of the cases in the previous panel. The Saddle-Node points are marked in blue. The output axis is displayed in log scale for visual clarity. Clear changes in the amplitude and ranges are shown. (**e**) Time series for the extreme ε values and the same input value.

The system of equations used is taken from a work by Tyson^2^ (see “Methods” section), where the parameter sets given are taken as a starting point. In Fig. 7a, we present a scheme of the model.

In the following, parameter b will be considered as the system ‘input’ and the activator x as the output. The input increases the growth rate of the inhibitor variable and will be used to draw the amplitude-frequency curves.

In Fig. 7b, we present the 2D bifurcation diagram, using ε and b as the scanning parameters. ε can control the underlying bistability and, as written in Tyson’s equations, is equal to the square root of the basal rate (the constant synthesis rate that remains when both variables are equal to zero). By manipulating said bistability, it fulfills the role of k_3_ in the DP oscillators.

As in the previous models, we find regions displaying oscillations, 3 FP excitability, and bistability, with codimension-two bifurcation points BT, CP, and GH. In this model, and with these parameter values, the BT and CP bifurcations are very close to each other, making clear the separation between all three regions. For high values of ε, a scanning of the input parameter determines oscillations between supercritical Hopf bifurcations. Below the GH point, one of the Hopf bifurcations is now subcritical, limiting the range of amplitude values as the system moves to that bifurcation. And once the subcritical Hopf bifurcations disappear, the oscillations are limited by SNIC bifurcations (magenta in Fig. 7b), contained within the Saddle-Node curve (blue in Fig. 7b). Here, the period can take much higher values, expanding the frequency range.

For low values of ε, the system can be either bistable or excitable (or monostable if the input is high enough). While the output is the variable that presents the positive feedback, the input is responsible for the production of the inhibitor. So, if the input takes low values, that favors the positive feedback over the negative one. This is consistent with the presence of a bistable region for low b and ε: the positive feedback dominates, and not only the self-activating output is bistable, but the two variable model is too. It is another example of a “transfer of bistability”^33^.

In Fig. 7c, we show four different amplitude vs. frequency curves, for different ε values and constructed with a scan of the input. We find similar results to the DP models, with a transition from an inverted-U curve to one with a wider range of frequencies as the system approaches the excitable and bistable zones (going from high to low ε), as well as lower values of frequency and higher of amplitude.

One important difference with previous results is the shape of the lowest-valued ε curve, which takes a loop resulting in two possible values for the amplitude within a certain range of the frequencies (three values when counting the low amplitude points, which are quite close to the supercritical Hopf bifurcation and at low values of parameter b). Already looking at the curve with the second lowest ε value (green curve in Fig. 7c), it is possible to note a certain path that the left branch of the curve takes: from low values of amplitude and a slight lowering of the frequency to higher values of frequency. This also happened in the 1 + 2 system, but in that case, the left branch transformed from a vertical one (low amplitude) to a horizontal one (low frequency) once the SNIC bifurcation became involved. Here, in the activator-inhibitor, instead of going “directly” to a region with low frequency, the curve forms a loop.

As the input increases in the scan, the system goes from a supercritical Hopf bifurcation to a SNIC, something that is quickly verified when looking at the 2D bifurcation diagram. In the 1 + 2 system, the path was inverted: it started at a SNIC and ended at a supercritical Hopf. This is due to the opposite effects of the inputs from both models: the 1 + 2 parameter went in favor of the output, while the activator-inhibitor one goes against it. However, when testing for an input that favors the output (multiplying the self-activating term with a “nu” parameter), the loop persisted. So, it is possible that the cause lays in how the equations are written (they do not follow mass-action kinetics, as in the DP systems). Nevertheless, the system still shows a wider range of frequencies due to the presence of the SNIC bifurcation, while having access to multiple amplitude values for the same frequency.

In Fig. 7d, we show the bistable curves for the isolated self-activating node, obtained by scanning the now parameter input y, and for each of the ε values used for the amplitude vs. frequency curves. It is clear how both the amplitude and the bistable range increase as ε decreases in value. That the lower stable steady state goes closer to zero as ε is lower is consistent with ε being the (square root of the) basal rate. And if the self-activating output resides at this lower state, the lower is ε, the lower the inhibiting input to transition to the upper state. This results in a wider bistable range. The connection with the results for the oscillator is clear. Lowering ε makes the positive feedback module show higher amplitudes and bistable ranges. Adding the negative feedback and decreasing the basal rate, the system will display a transition in the types of oscillations obtained, eventually crossing over to an excitable or bistable region.

In Fig. 7e, we present a pair of oscillating time series for the extreme values of ε, at the same input value (b = 0.14). There are evident differences between both outputs. While the series for low ε has a high-amplitude spike-like form, the other one looks more like a small sinusoidal wave, with a higher amount of peaks in the same time window. These two cases are close to the boundaries of the oscillatory region and, as such, represent opposite ends of the spectrum of outputs. It is also possible to fix, for example, ε at 0.065 and obtain very different frequencies, while slowly modifying the input parameter close to the SNIC bifurcation. The results obtained via bifurcations and amplitude vs. frequency curves allow to tinker with the parameters to obtain a desired result.

It is worth noting that the two parameters selected for the bifurcation analysis control one feedback each: ε acts on the positive feedback, modifying the bistable amplitude and range, while the input parameter b acts on the negative loop, by regulating the production of the inhibitor variable. In a model like the activator-inhibitor, this selection of parameters is made easier due to the relative simplicity of the network.

An aspect that has not been approached in this model is the timescale separation between the variables. The parameter τ governs the timescale (see equations in “Methods” section), and we fixed τ = 10. It is possible to lower it and still obtain similar results. But for a low enough value, the ε vs. b diagram will only display Hopf bifurcations (see Supplementary Fig. S6). This is consistent with the results for the SK model when k_i,K_ is close to 1 (Supplementary Fig. S4). So, as in the SK model, there is another path of analysis for the activator-inhibitor that can be combined with the control of the underlying bistability.

The results presented in this section show how to approach an oscillator with a bistable module different from the one in previous sections. If there is a parameter capable of manipulating the underlying bistability, it should be possible to find a spectrum of behavior in the oscillatory outputs. It could be a relatively complex system like the DP cycle, or something more direct like a self-activating output. Our methods could be applied to other examples of oscillators with bistable subsystems.

## Discussion

The concept of feedback loop is preponderant in the analysis of the fundamental mechanisms which govern the existence of a system’s temporal oscillations^3^. The identification of these feedback loops can be easy, as in many biological models of only two components (e.g., activator-inhibitor, prey-predator, etc.). Other times, with more than two components, the positive or negative feedbacks are still linked to well-identified physical mechanisms (e.g., in conductance-based models^11^). But in certain biological systems, the identification of feedbacks is not completely self-evident. Here, the analysis of temporal oscillations is all the more interesting, as it is intimately linked to some features of the system which at the start are somewhat veiled.

Specifically, this may be the case in biochemical networks (e.g., signaling pathways, genetic networks, or metabolic networks), represented graphically in multiple ways in the literature, so that the arrows and other symbols used in these graphs sometimes have very different meanings: activation, transport, binding, simple chemical transformation, enzymatic reaction, etc. These graphic representations can be very useful for diagramming cause-effect relationships in these systems, and in particular, for highlighting simple but essential feedback loops in the behavior of the system. On the other hand, these systems can also contain *implicit* (or *non-explicit*) feedback loops which are not obvious in the biochemical diagram, but nevertheless play an essential role in the understanding of behaviors. For example, the vast majority of signaling pathway diagrams contain negative feedback loops that are implicit^31,36,37^. From a biochemical point of view, it is relatively simple to identify in an enzymatic reaction a “negative” interaction associated with enzymatic sequestration^38^. But highlighting a whole negative feedback loop in the biochemical network requires a deeper analysis, which is not necessarily intuitive. This is what we have achieved, and which gives an interesting facet to our work, where the analysis of the temporal oscillations exhibited by the signaling patterns we have studied is intimately linked to the existence of these implicit feedbacks.

In this article we consider a double phosphorylation cycle, a ubiquitous signaling component, having the ability to display bistability^24^. The existence of more than one steady state under particular biological conditions is strongly related to the existence of positive feedback loops, as was shown first for gene regulatory networks^39^ and then demonstrated as a necessary condition^40^. If this component, characterized by positive feedback loops and emergent multistability, is connected to other signaling elements, it very likely undergoes some sort of protein–protein interaction, reported to generate hidden feedbacks^36^. Depending on the particular signaling scenario and on the authors, the effect of these connections were termed sequestration^31^, retroactivity^37^, effect of shared components^28^, interactions with elements of a more extended network^41^, substate-dependent control^25^, load-induced modulation^42^, off-target effects^43^ or competition effects^44^.

In several cases, these protein–protein interactions result in a non-explicit negative feedback effect, leading to interlinked positive and negative feedbacks. This combination was studied in the literature as a way to generate relaxation-type oscillations^8,23,25^. Here, we showed that the two feedbacks together ensure two types of oscillations, the relaxation-type ones and a smoother type of oscillations functioning in a very narrow range of frequencies, in such a way that outside that range, the amplitude of the oscillations is severely compromised. Even more, we showed that the two feedbacks are essential for both oscillatory types to emerge, and it is their hierarchy what determines the type of oscillation at work.

Other studies in the literature deal with different types of oscillations, but since they add a positive feedback to a system already capable of oscillatory behavior, like three nodes with delay^15,19,45^, they differ from the double phosphorylation cycle models analyzed in our work. As mentioned before for the two- or three-level cascades, the positive feedback present in the double phosphorylation cycle, that leads to bistability in said cycle, is essential for oscillations^23^.

**Figure 8:**
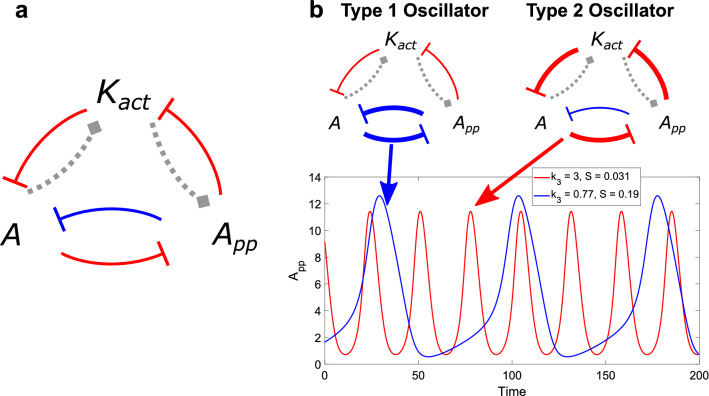
Network of the feedbacks between the variables K_act_, A and A_pp_ of the reduced Suwanmajo-Krishnan signaling motif. (**a**) In red, the three negative interactions that constitute the negative feedback loop. The blue and red interactions between A and A_pp_ make up the positive feedback loop. In grey, the interactions than can be either positive or negative. (**b**) The two possible types of oscillator, depending on the relative strength between the feedbacks. This strength is modulated by the biochemical parameters. For example, when k_3_ is relatively low, the oscillations will be of the relaxation-type (bold blue for the positive feedback loop, blue time series). When k_3_ is high, the output will be smooth and symmetrical (bold red for the negative feedback loop, red time series).

In the first two sections, we studied different models based on said double phosphorylation cycle, a ubiquitous motif in cell signaling. In our studies, this cycle is activated either by a 2-state kinase (active or inactive) or by a single-phosphorylated cycle. In both cases, although the feedback structure of the signaling components is not explicit in the biochemical scheme, by using a quasi-steady approach, the initial system can be reduced to a 3-variable network revealing that the underlying interaction graph is characterized by two interlinked positive and negative feedback loops (see Fig. 8a, Supplementary Methods for the computation of the interaction graph). Moreover, in the studied cases, the subsystem corresponding to the DP cycle displayed bistability. Therefore, our oscillator models can be represented by a nested bistable module within a negative feedback loop. Selecting parameters capable of manipulating the underlying bistable subsystem, we identified the corresponding bifurcations when scanning pairs of relevant parameters. Furthermore, we analyzed the output of the oscillators in terms of their amplitudes and frequencies when moving the input.

The first model analyzed, designed by Suwanmajo and Krishnan, is the simplest oscillator based on a bistable double phosphorylation cycle driven by a 2-state kinase. First, we analyzed the changes of oscillation characteristics caused by the timescale difference between the activation of the kinase and the rest of the system. This displayed the possibility to obtain different types of oscillations while leaving the underlying bistability intact. The ranges and values for the amplitude and frequency of the oscillatory output greatly depended on the choice of timescale. Meaning, the existence of the inactive state for the kinase is not only responsible for the emergence of oscillations^26^, but it opens up the system to varying outputs by controlling the speed of the activation module reactions.

For relaxation oscillations, it can be usual to encounter models with differing timescales in the variables. However, since we are working with models where bistability is critical to provide oscillations, we proceeded to manipulate said bistable behavior present in the subsystem while keeping a moderate timescale separation. We wanted to show that a relatively high degree of timescale separation is not necessary to obtain a variety of oscillatory outputs.

In the SK model, the parameter involved in the first phosphorylation step for the substrate, the k_3_ parameter, is of particular importance. It influences how the free active kinase will react in the DP cycle, and how much of it is available for inactivation in the first level. Our results for the scanning of k_3_ and the input S showed significant differences with the timescale separation studies. A co-dimension 2 Bogdanov–Takens bifurcation was obtained, resulting in particular in Saddle-Homoclinic and Saddle-Node bifurcations.

The first one, which is of the global type, has a drastic effect on the amplitude of the oscillations, while the second one, a local bifurcation, gives way to a region of bistability in the system. The dynamical map became richer than when only the timescale was changed, while showing that a large timescale separation is not essential to obtain different types of oscillatory outputs.

When adding another control parameter from the DP cycle, namely P_tot_, it becomes clear how much potential the bistable subsystem has in determining the output oscillations. While k_3_ modifies the bistable range, P_tot_ works on the amplitude. With both parameters, one can influence the amplitude and frequency of the output in the same system. It is worth mentioning that this type of manipulation does not need to be restricted to our parameter choice. Other rate constants, and even the total amount of substrate, could also have a noticeable effect on the oscillations. At the same time, we aimed to show how from previously used parameter values^26^ it is possible to find different types of oscillations. Our analysis works in the regions of parameter space that we explored and can be extended to others where the underlying bistability is present. Studies like Ref.^46^ can be used to probe and analyze new bistable regions of the DP cycle. In all, our results show that with just a few parameter choices and values, a spectrum of oscillatory behaviors can arise.

As mentioned, the parameter k_3_ was key in interpreting the results, since it controls how much the free active kinase is sequestered in each level: how much is taken for deactivation in the first level and how much is used to form an intermediate complex with the unphosphorylated substrate in the second level. k_3_ modulates the relative strengths of the nested positive and negative feedback loops schematized in Fig. 8b, and thereby enables to switch from one oscillatory type exhibited by the signaling motif to another.

The first model studied considered that the activation and the inactivation of the first-level kinase were respectively performed in one step. A natural extension of this model was the case where the first-level kinase was subject to a single phosphorylation cycle. In this sense, the second model was equivalent to a MAPK cascade restricted to its two first stages. This allowed us to analyze a two-level cascade with added complexity, showing that the previously applied methodology can still result in different types of oscillations. While the general picture obtained from analyzing this so-called 1 + 2 model was similar to the direct activation case, there were marked differences.

The bifurcation analysis showed a wider oscillatory region than for the Suwanmajo–Krishnan model, with the same values for every fixed DP parameter. The increased oscillatory range for k_3_ was the main feature of this change, even though the underlying bistable range for k_3_ remained the same. The added reactions in the first level allow the system to take further advantage of the DP cycle bistability. For instance, SNIC bifurcations were found, which allowed for the frequency to be considerably lowered. This was not possible in the SK model, where the parameter values selected displayed LPC bifurcations close to the SHom bifurcations, preventing the stable limit cycles to increase their period. While the SNIC bifurcation allows a wider frequency range, the prevalence of supercritical Hopf bifurcations leads to smaller amplitude values, seen in the different amplitude vs. frequency curves. In all, the 1 + 2 model displays significant enhancements from the minimal model previously studied.

At the same time, since manipulating the bistability of the DP module allows for a wide spectrum of periodic outputs, it would be interesting to explore other sequestration possibilities. We concentrated on the kinase, but as mentioned, Suwanmajo and Krishnan also present a minimal model with phosphatase activation and inactivation. One could also sequester the output with a single phosphorylation cycle (a “2 + 1” model) and investigate the new output in that single cycle for different bistable sets of the DP level. Our results can constitute a guide for the analysis of numerous oscillators based on a bistable double phosphorylation cycle. Starting from that module in isolation, the extended systems will respond accordingly.

To extend our analysis beyond oscillators based on a DP cycle, we studied an example of the activator-inhibitor model, where the underlying bistability comes from a simple auto-catalytic step. Once again, we took a parameter capable of manipulating said bistability and performed a scan of it versus an input. While the input parameter acted in a way opposite to that of the DP systems, and the differential equations were not written following mass-action kinetics, the results obtained were similar to the previously studied models. As in the 1 + 2 case, SNIC bifurcations were found in the activator-inhibitor, allowing the period to increase significantly. The amplitude vs. frequency curve scanned near the bistable region displayed a new type of shape, that was not met in the DP cycle models.

All three models studied were capable of producing a variety of oscillations, with varying ranges and values for the amplitude and the frequency. The bifurcation analysis showed the possibilities of transitions from oscillations to bistable and excitable regimes. Since the three models are capable of oscillatory behaviors due to underlying bistability, it follows that they can enter these other regimes for the appropriate parameter values. If said module bistability is strong enough, SN bifurcations will appear in the full systems, developing a bistable region. Also, Hopf bifurcations can destabilize one of two stable steady-states, giving way to an excitable output.

In particular, this excitable region found in our work can be of potential interest. As far as we know, there are very few studies concerning the idea of excitability in signaling systems^47–49^, as opposed to bistability and oscillations, so our results could be used to study this behavior in more detail.

Furthermore, our focus on the response to input parameters could prove particularly useful when analyzing an experimental model. In a work from 2010 by Tigges et al.^50^ they study such a model, presenting a positive feedback loop embedded within a negative one. Since it appears that the subsystem corresponding to the positive loop displays bistability, this experimental model is a representative example of the systems studied in our work. And being constructed for a circadian clock, its frequency should reside within a very small range. As we have shown, underlying bistability can drive a system to global bifurcations where the period diverges (as well as push it to a bistable or excitable regime).

Using the reference parameter set given by the authors, analyzing the two variables that constitute the positive feedback loop, and taking the input variable as a parameter, we found that a bistable range emerges. Also, one of the system parameters is capable of controlling said range. From this, by scanning only two parameters, we were able to locate SHom bifurcations, leading to different amplitude vs. frequency curves like the ones shown for the three models previously analyzed (see Supplementary Fig. S7 for the results on this model).

These results show how our methods can be applied to an experimental case. By showing a path in parameter space to unwanted outputs (tunable frequency in a circadian clock), our findings point out the importance of appropriately setting the parameter values to obtain an output consistent with the data. The existence of a nested bistable module in an oscillator that must display fixed frequencies should be taken into careful consideration.

The oscillatory behaviors we found for the SK and 1 + 2 systems will serve, in a following study, as a starting point for the analysis of the complete MAPK cascade and its potential different outputs. The addition of a second DP cycle will introduce another level capable of bistable behavior, although it is not necessary for both DP modules to be bistable to obtain cascade oscillations^23^. Studying how the amplitude and period of the oscillations depend on the different parameter combinations for the two DP cycles will constitute another avenue of analysis.

Our study points out the wealth of oscillatory dynamics that exists in a system consisting of a bistable module nested within a negative feedback loop. It shows how to transition between different types of oscillations and other dynamical behaviors such as excitability, while providing a framework to study other systems like the three level MAPK cascade. Furthermore, our results can be used to analyze other types of bistable modules, since it works on very different cases like the DP cycle and an auto-catalytic node, plus a double positive interaction case^50^. A simple phosphorylation cycle with translocation between compartments has been studied for its bistability^51^, and adding sequestration could result in oscillations (like it occurs for the DP cycle). And a 2018 work by Perez-Carrasco et al. shows the behavior that arises from combining a toggle switch and a repressilator, that is not found when both modules are separated^52^. Manipulation of the bistable switch could be done to investigate different oscillatory outputs. Basically, any oscillatory system with a bistable subsystem is a potential new candidate for our type of study. Controlling that underlying bistability can lead to the kind of results presented in this work.

At the same time, our analysis could be useful for experimental cases. The 2014 work by Tsai et al. on a cell cycle system shows the importance of analyzing different oscillatory types in a computational model, since it explains experimental findings^14^. And a recent work on the cell cycle model investigates the oscillatory robustness as a function of the cytoplasmic density, where they find limit cycles forming a hysteresis loop^53^. Simulations explain this through subcritical Hopf bifurcations, showing the significance of dynamical studies on experimental examples. Changes in the period are also found, opening a potential avenue of analysis for the amplitude and the frequency. In all, our work could provide a map to both understand real life data and predict new behavior in biological oscillators.

## Methods

### MATCONT and MATLAB

For the bifurcation analysis we used the software MATCONT^54^, a graphical MATLAB software package for the interactive numerical study of dynamical systems. After finding the steady-state, the algorithm performs a continuation analysis while changing the parameter of choice to build a bifurcation diagram.

For the time series and amplitude vs. frequency curves, we used MATLAB (Statistical Toolbox, version 2017b, The MathWorks, Inc., Natick, Massachusetts, United States). The ODE solver was ode15s (also used in MATCONT), with relative and absolute tolerances of 10^–6^ and 10^–8^ respectively to obtain trustworthy numerical results. These tolerance values were lowered in a few cases where deemed necessary (e.g., where the system analyzed was very close to a SNIC).

For the amplitude vs. frequency curves, we first used in MATLAB the Fast Fourier Transform (fft) to discriminate oscillatory cases. If the series was oscillatory, it was run again using the last point of the previous simulation as the first point of the new simulation, to leave aside any possible transitory oscillations and have the final oscillatory output over the length of the series. This new time series was used to calculate the amplitude and frequency, by finding the peaks and valleys in the series. Calculating the output difference between each peak and its neighboring valley, an average of the differences was computed for the amplitude. And using the different intervals of time between peaks, the average of said intervals resulted in the period. The length of time for each simulation was determined so as to have several peaks for the averages while also trying to limit the computation time (shorter periods get shorter lengths of time).

### Model equations

The equations for the Suwanmajo-Krishnan model are taken from their work^26^. The equation for the inactive kinase (the last of the following system) is rewritten to show the control of the timescale separation done by the first-level constants (k_c_ = 1 in all cases studied).

1 $$\begin{array}{*{20}l} {\frac{d\left[ A \right]}{{dt}} = k_{2} \left[ {AK} \right] - k_{1} \left[ A \right]\left[ K \right] + k_{12} \left[ {A_{p} P} \right],} \hfill \\ {\frac{d\left[ K \right]}{{dt}} = (k_{2} + k_{3} )\left[ {AK} \right] - k_{1} \left[ A \right]\left[ K \right] + (k_{5} + k_{6} )\left[ {A_{p} K} \right] - k_{4} \left[ {A_{p} } \right]\left[ K \right] - k_{i,K} \left[ K \right] + k_{a,K} S\left[ {K_{0} } \right],} \hfill \\ {\frac{{d\left[ {AK} \right]}}{dt} = k_{1} \left[ A \right]\left[ K \right] - (k_{2} + k_{3} )\left[ {AK} \right],} \hfill \\ {\frac{{d\left[ {A_{p} } \right]}}{dt} = k_{3} \left[ {AK} \right] - k_{4} \left[ {A_{p} } \right]\left[ K \right] + k_{5} \left[ {A_{p} K} \right] + k_{9} \left[ {A_{pp} P} \right] + k_{11} \left[ {A_{p} P} \right] - k_{10} \left[ {A_{p} } \right]\left[ P \right],} \hfill \\ {\frac{{d\left[ {A_{p} K} \right]}}{dt} = k_{4} \left[ {A_{p} } \right]\left[ K \right] - (k_{5} + k_{6} )\left[ {A_{p} K} \right],} \hfill \\ {\frac{{d\left[ {A_{pp} } \right]}}{dt} = k_{6} \left[ {A_{p} K} \right] + k_{8} \left[ {A_{pp} P} \right] - k_{7} \left[ {A_{pp} } \right]\left[ P \right],} \hfill \\ {\frac{d\left[ P \right]}{{dt}} = (k_{8} + k_{9} )\left[ {A_{pp} P} \right] - k_{7} \left[ {A_{pp} } \right]\left[ P \right] + (k_{11} + k_{12} )\left[ {A_{p} P} \right] - k_{10} \left[ {A_{p} } \right]\left[ P \right],} \hfill \\ {\frac{{d\left[ {A_{pp} P} \right]}}{dt} = k_{7} \left[ {A_{pp} } \right]\left[ P \right] - (k_{8} + k_{9} )\left[ {A_{pp} P} \right],} \hfill \\ {\frac{{d\left[ {A_{p} P} \right]}}{dt} = k_{10} \left[ {A_{p} } \right]\left[ P \right] - (k_{11} + k_{12} )\left[ {A_{p} P} \right],} \hfill \\ {\frac{{d\left[ {K_{0} } \right]}}{dt} = k_{i,K} \left[ K \right] - k_{a,K} S\left[ {K_{0} } \right] = k_{i,K} \left( {\left[ K \right] - k_{c} S\left[ {K_{0} } \right]} \right).} \hfill \\ \end{array}$$

The equations for the 1 + 2 model are the same as for the SK model, with new terms for the active kinase, the new equation for the inactive kinase of the first level, and the addition of the four new variables.

2 $$\begin{array}{*{20}l} {\frac{d\left[ K \right]}{{dt}} = (k_{2} + k_{3} )\left[ {AK} \right] - k_{1} \left[ A \right]\left[ K \right] + (k_{5} + k_{6} )\left[ {A_{p} K} \right] - k_{4} \left[ {A_{p} } \right]\left[ K \right] + a_{3} \left[ {K_{0} E_{1} } \right] - a_{4} \left[ K \right]\left[ {E_{2} } \right] + a_{5} \left[ {KE_{2} } \right],} \hfill \\ {\frac{{d\left[ {K_{0} } \right]}}{dt} = - a_{1} \left[ {K_{0} ][E_{1} } \right] + a_{2} \left[ {K_{0} E_{1} } \right] + a_{6} \left[ {KE_{2} } \right],} \hfill \\ {\frac{{d\left[ {K_{0} E_{1} } \right]}}{dt} = a_{1} \left[ {K_{0} ][E_{1} } \right] - (a_{2} + a_{3} )\left[ {K_{0} E_{1} } \right],} \hfill \\ {\frac{{d\left[ {KE_{2} } \right]}}{dt} = a_{4} \left[ {K][E_{2} } \right] - (a_{5} + a_{6} )\left[ {KE_{2} } \right],} \hfill \\ {\frac{{d\left[ {E_{1} } \right]}}{dt} = - a_{1} \left[ {K_{0} ][E_{1} } \right] + (a_{2} + a_{3} )\left[ {K_{0} E_{1} } \right],} \hfill \\ {\frac{{d\left[ {E_{2} } \right]}}{dt} = - a_{4} \left[ {K][E_{2} } \right] + (a_{5} + a_{6} )\left[ {KE_{2} } \right].} \hfill \\ \end{array}$$

Finally, the equations for the activator-inhibitor model are taken from Tyson’s chapter^2^.

3 $$\begin{array}{*{20}l} {\frac{dx}{{dt}} = \frac{{\varepsilon^{2} + x^{2} }}{{1 + x^{2} }}\frac{1}{1 + y} - ax,} \hfill \\ {\tau \frac{dy}{{dt}} = b - \frac{y}{{1 + cx^{2} }}.} \hfill \\ \end{array}$$

### Parameter values

As mentioned in the “Discussion” section, we aimed to show how from previously used parameter values^2,26^ it is possible to find different types of oscillations. These sets from previous works were the starting point for the different explorations that we did in the bifurcation analyses and amplitude vs. frequency measures. Our analysis works in the regions of parameter space that we explored and could be extended to others where the underlying bistability is present.

The legends within panels provide the values changed when plotting different cases (amplitude vs. frequency curves, limit cycles, bistable curves, and time series). The rest of the information is below.

#### SK model

Unless specified otherwise in the below information, the parameter values are: A_tot_ = 40, K_tot_ = 17, P_tot_ = 17, k_1_ = 8, k_6_ = 50, all other rate constants equal to 1. This also applies to the parameters of the DP cycle when showing its bistable curves.

Figure 2: (a) scan of k_a,K_ = k_i,K_ vs. S; (b) scan of S for different k_a,K_ = k_i,K_ cases; (c) S = 0.1 for different k_a,K_ = k_i,K_ cases; (d) see plot legend.

Figure 3: k_a,K_ = k_i,K_ = 0.65 in all panels. (a), (b) scan of k_3_ vs. S; (c) scan of S for different k_3_ cases; (d) scan of S for k_3_ = 0.77; (e) scan of K_act_ in the DP cycle for different k_3_ cases; (f) see plot legend.

Figure 4: k_a,K_ = k_i,K_ = 0.65 in all panels. (a) scan of k_3_ vs. P_tot_ with S = 0.1; (b) scan of S for different k_3_ and P_tot_ cases; (c) scan of K_act_ in the DP cycle for different k_3_ and P_tot_ cases; (d) see plot legend.

#### 1 + 2 model

All DP cycle parameters have the same values as before unless specified otherwise (different values of k_3_ and P_tot_). All rate constants in the first level are equal to 1. E_2tot_ (total amount of phosphatase in the first level) is equal to 1.

We chose 1 as the values for the new rate constants, following the SK set of parameters. Not only many of the rate constant values from SK were equal to 1 in the DP cycle, but our choice also put the parameter values of the first level in the same order of magnitude as the activation and inactivation constants from the SK model. Our goal was to obtain an oscillatory set that did not differ significantly from the original SK set, to make the comparison in results between the motifs more robust.

Figure 6: (a), (b) scan of k_3_ vs. P_tot_ with E_1tot_ = 0.21; (c) scan of E_1tot_ for different k_3_ and P_tot_ cases; (d) scan of K_act_ in the DP cycle for different k_3_ and P_tot_ cases; (e) see plot legend.

#### Activator-inhibitor model

a = 0.1, c = 100 and τ = 10 in all cases, as used in Ref.^2^.

Figure 7: (b) scan of ε vs. b; (c) scan of b for different ε values; (d) scan of y in the auto-catalytic node for different ε values; (e) see plot legend.

### SBML files

We provide SBML files (Systems Biology Markup Language) for each of the models studied in this work. These were created using the software MOCCASIN^55^.

## Supplementary Information

Supplementary Information.

## Data Availability

The datasets used and/or analyzed during the current study are available from the corresponding authors on reasonable request.
