# Supplementary material for: A nested bistable module within a negative feedback loop ensures different types of oscillations in signaling systems
Source: Sci Rep. 2023 Jan 11;13:529. doi: 10.1038/s41598-022-27047-4 (PMC9834387; doi:10.1038/s41598-022-27047-4)
Supplement: Supplementary file 1 — Supplementary Information. [file 41598_2022_27047_MOESM1_ESM.pdf]

A nested bistable module within a negative feedback loop ensures different types of oscillations in signaling systems

Supplementary Information

Juan Ignacio Marrone, Jacques-Alexandre Sepulchre, Alejandra C. Ventura

Supplementary Figures

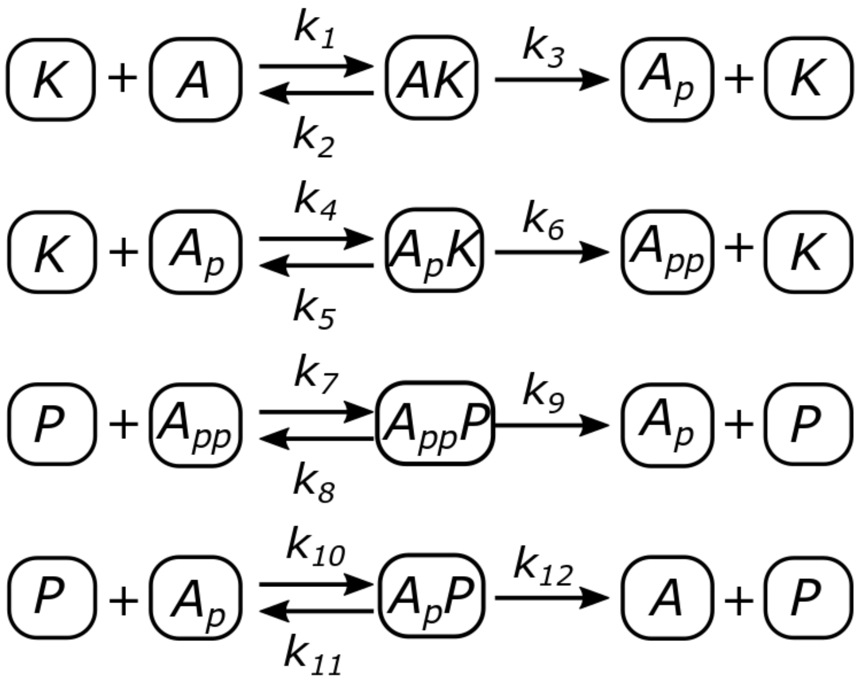

Figure S1: Scheme of the reactions in the DP cycle. These reactions take place both in the SK model and the 1+2 model.

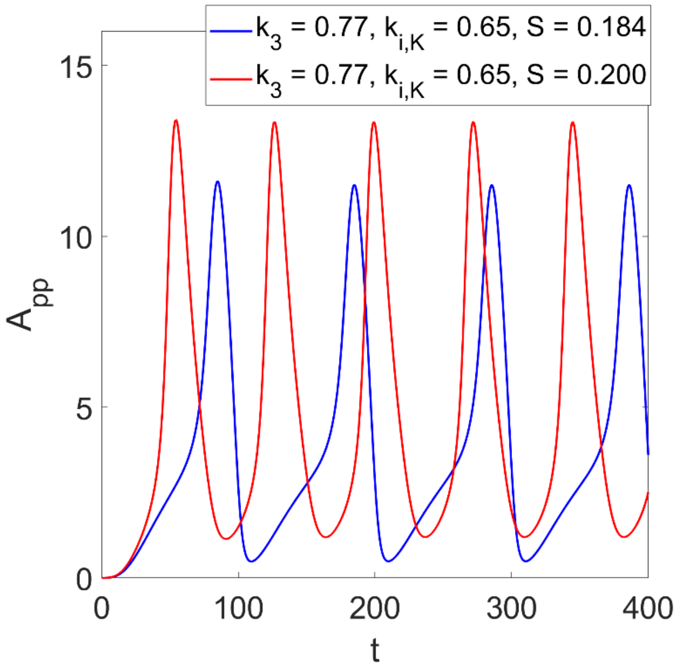

Figure S2: Temporal series for the SK model, at k_3=0.77 and for two different values of the input parameter S. While the case S=0.200 displays a relatively symmetrical shape, the case with lower input and closer to the SHom bifurcation is asymmetrical. This shows an alternative way to obtain different oscillations, not only changing a parameter like k_3, but exploiting the different bifurcations present in the system when a scan of the input is performed.

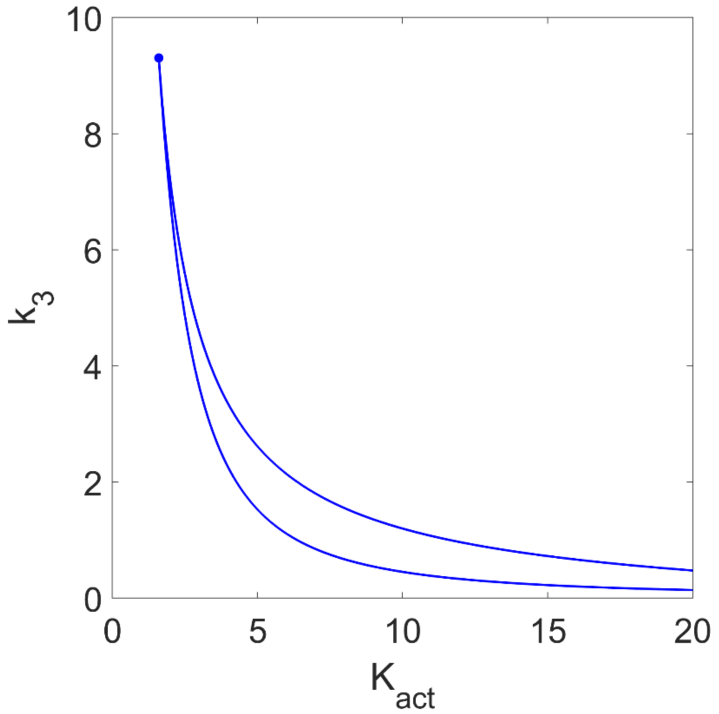

Figure S3: 2D bifurcation diagram in the DP cycle scanning k_3 and K_{act}. All points on the blue curves are Saddle-Nodes. The blue point on the end is a Cusp Point (at k_3=9.305 approx.). The region defined is bistable. All other parameters take the same values as the ones used for the SK and 1+2 models (k_1=8, k_6=50, A_{tot}=40, P_{tot}=17, every other k=1). It is expected that for values of k_3 higher than the Cusp Point, the SK and 1+2 models will not display oscillations.

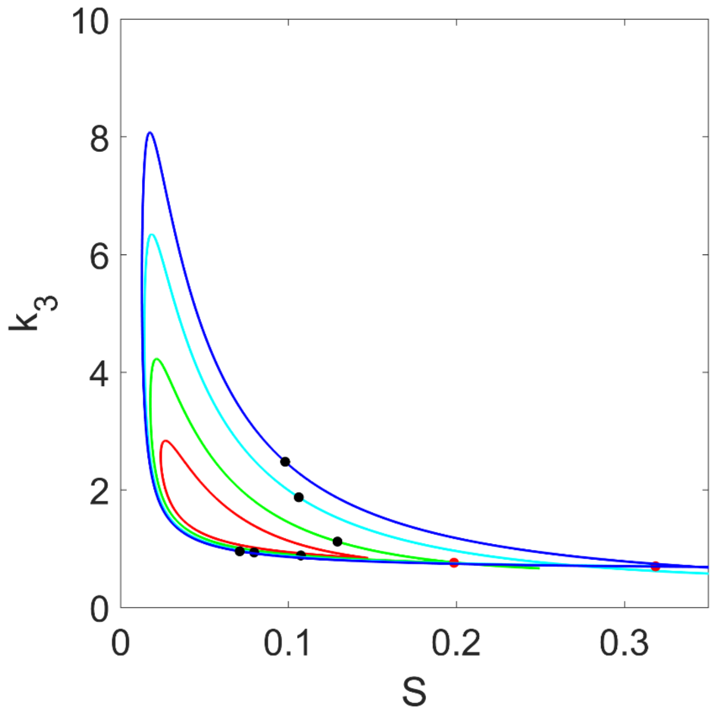

Figure S4: 2D bifurcation diagram for the SK model, when scanning k_3 and S. All curves are Hopf. The $k_{a,K}$=$k_{i,K}$ cases shown are: 1 (in red), 0.65 (in green), 0.29 (in cyan), 0.10 (in blue). Black points are GH bifurcations, red one are BT. As the timescale separation increases, the oscillatory region increases, and in particular, the range of k_3 widens. When comparing with Fig. S3, it is possible to see that the top of the region gets closer to the Cusp Point found in the DP cycle, but it does not overtake it. Increasing the timescale separation allows the SK model to take more advantage of the underlying bistability, while being limited by it to values below the Cusp Point. At the same time, when $k_{a,K}$=$k_{i,K}$ is higher, only Hopf bifurcations are found. In the lowest timescale separation case (red curve), not even GH bifurcations are found and all Hopf points are supercritical.

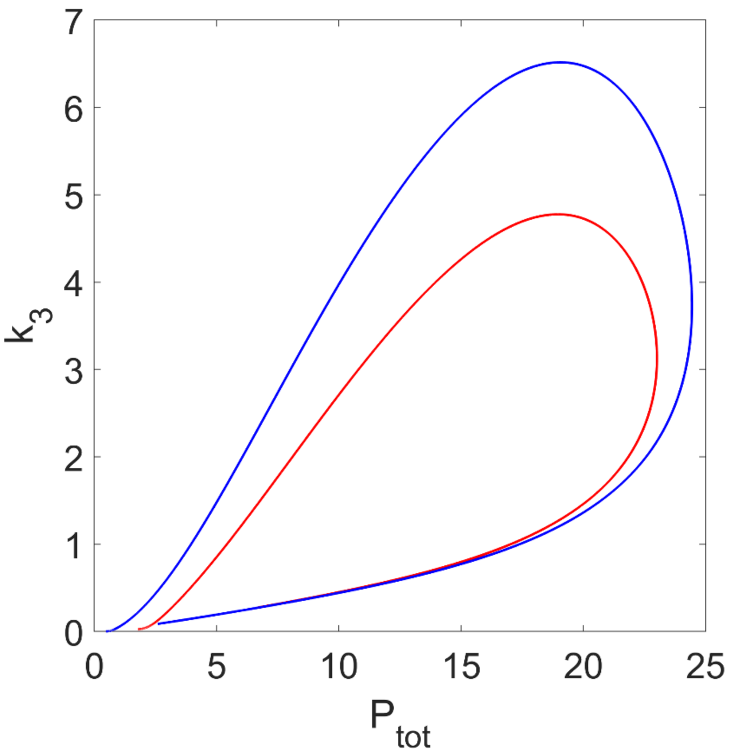

Figure S5: 2D bifurcation diagram for the 1+2 model, showing only the Hopf curves. The red case corresponds to all the rate constants in the first level being equal to 1. The blue case, all equal to 0.1. This lowering of the first-level values is analog to the study performed in the SK model when changing the timescale separation through the direct activation and inactivation rates. Once again, the oscillatory region expands when increasing the timescale separation.

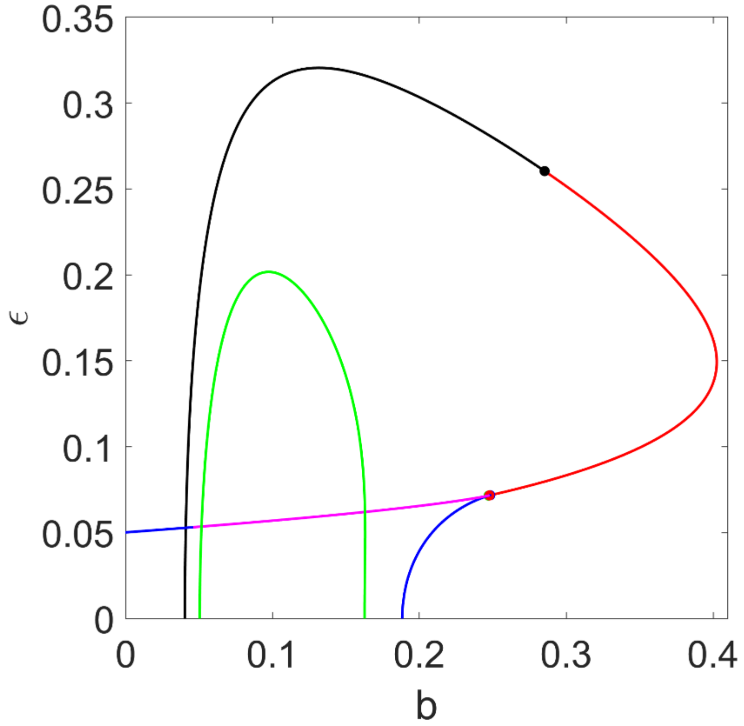

Figure S6: 2D bifurcation diagram for the Activator-Inhibitor model. The green curve corresponds to Hopf bifurcation at τ=1. The other curves are the same as the ones shown in the main text, with τ=10. When the timescale separation is lower (τ=1), only Hopf bifurcations are found, as was the case for the SK model (see Fig. S4, red curve).

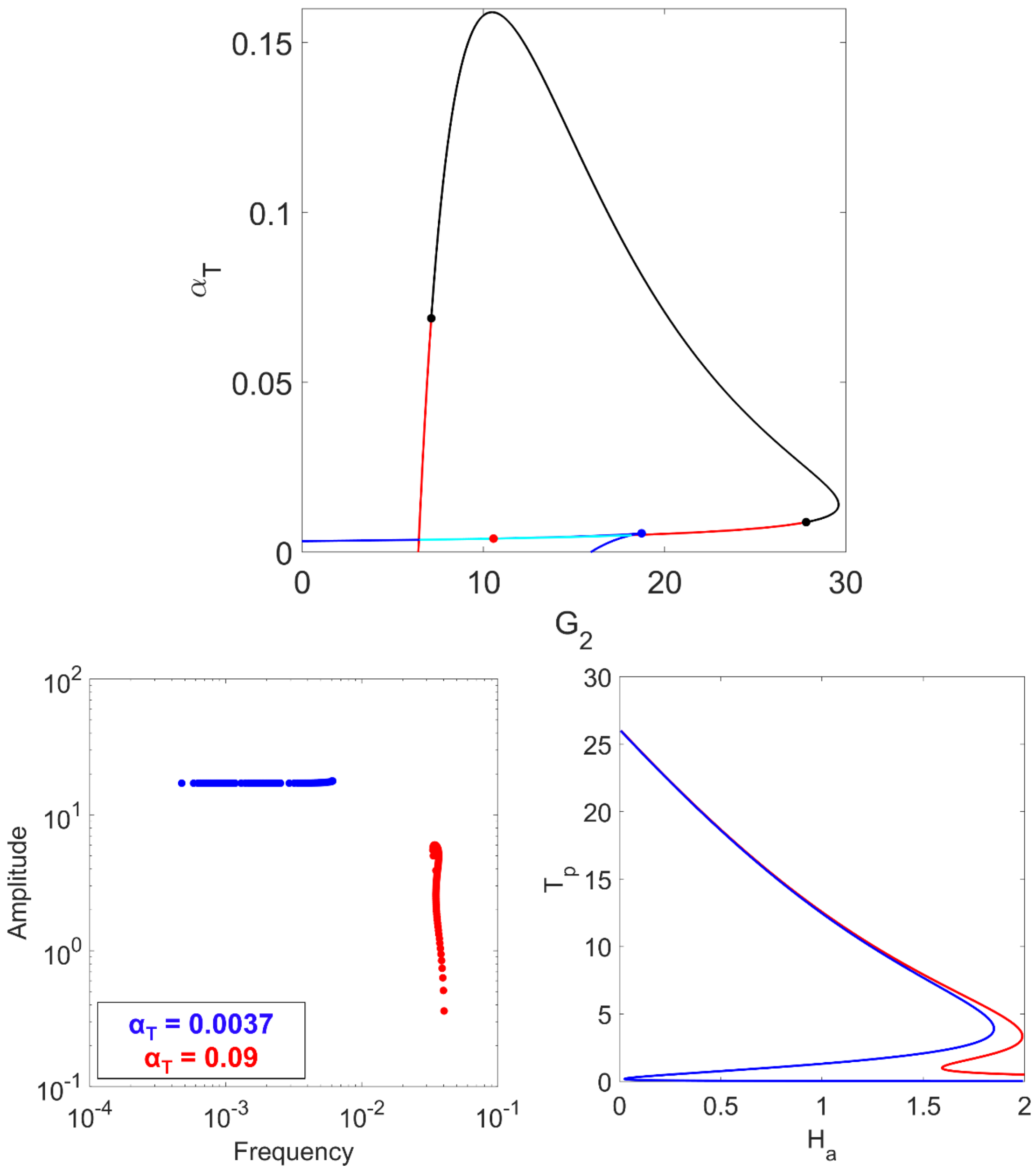

Figure S7: Results for the Tigges et al. model. Top: 2D bifurcation diagram for α_T (capable of controlling the underlying bistability) versus G_2 (input parameter). Supercritical Hopf curve in black, subcritical in red, SN curve in blue, SHom curve in cyan. GH bifurcations in black, CP in blue, BT in red. Fixed parameters changed with respect to the reference set given by the authors: G_1=10, k_{DTp}=0.02426. The bifurcation diagram displays similar characteristics to the ones shown in the main text, with supercritical Hopf bifurcations to high values of α_T and both SN and SHom bifurcations below. Lower left: amplitude vs. frequency curves for α_T=0.0037 (in blue) and 0.09 (in red, reference value), scanning the input G_2. Clear contrast between the shapes, going from a fixed frequency and tunable amplitude at 0.09 to the opposite behavior at 0.0037. The scan is quite precise as the input approaches the SHom bifurcation. Lower right: underlying bistable curves for the two cases studied in the previous panel, with a marked increase in the width of the bistable range when α_T=0.0037.

Supplementary Methods

In this section we show that, in the framework of the quasi-steady approximation, the dynamics of the Suwanmajo-Krishnan model [1] can be reduced to a system of only 3 coupled variables. Then we show that, similarly, the dynamics of the first two layers of the MAPK cascades can be reduced to a 3-variable system [2, 3]. Finally we will show that the underlying interaction graph of each of these reduced models can be described by 2 interlinked positive and negative feedback loops.
Let us start with the Suwanmajo-Krishnan model and afterwards the same analysis will apply to the truncated MAPK signaling cascade.

Dimension reduction of the Suwanmajo-Krishnan model

The Suwanmajo-Krishnan (SK) model describes a signaling motif formed by a double phosphorylation (DP) cycle whose enzymes, kinase and phosphatase, are subjected to activation/deactivation reactions. In the following, as in the main text of this paper, we consider only the activation of the kinase.
The equations of the model are taken from Appendix A of the paper [1] and we adopt the same notations. In the original SK model there are 11 coupled equations. Since we consider only the activation of the kinase, not the phosphatase, the system reduces here to 10 coupled equations. Using 3 conserved quantities (total kinase K_{tot}, total phosphatase P_{tot}, and total protein A_{tot}), the phase space has 7 dimensions.
The system can be decomposed as formed by 2 modules:
(1) the DP cycle (called the "isolated system" by the authors),
(2) the activation module. The activation is a reversible reaction playing the role of a switch between the inactive state and the active state of the kinase.
A well-known method to reduce the number of variables is to invoke the quasi-steady state approximation for the complexes (enzyme-substrate) [4]. In this framework the variables describing the concentrations of these complexes are taken at their equilibrium values. Since there exist 4 such complexes, one can reduce the system dynamics to a system of 7-4=3 coupled equations.
The DP module can then be described by 2 independent variables that we choose as A and A_{pp}. The single-phosphorlylated species A_p can be deduced from the conservation of the total protein A_{tot}.
The activation module can be represented by the *total activated kinase*, that is (using the notation of the SK paper) : $K_{tot} - [K_0] = [K] + [AK] + [A_pK]$. Let us name it K_a. So, in the following $K_a = [K] + [AK] + [A_pK]$. Then, by summing $\frac{d}{dt}([K] + [AK] + [A_pK])$ in the equations (A1) of the Appendix A of the paper, most of the terms cancel each other, and one readily finds:

$$\dot{K}_a = -k_{i,K}[K] + k_{a,K}S_1[K_0] \tag{1}$$

In this equation it is straightforward to replace $[K_0]$ by $K_{tot} - K_a$. On the other hand, using the detailed analysis in section A.2 of the Appendix of [1] one gets:

$$K_a = [K] + [AK] + [A_pK] = [K] + c_1[A][K] + c_2[A_p][K] \tag{2}$$

This latter equation enables to replace $[K]$ in function of K_a, A and A_{pp} in eq.(1). In eq.(2), the parameters c_1 and c_2 are defined by Suwanmajo and

Krishnan as $c_1 = k_1/(k_2 + k_3)$ and $c_2 = k_4/(k_5 + k_6)$. They correspond to the inverses of the traditional Michaelis-Menten constants. In the following we will stick with their notations and define also the other coefficients $c_3 = k_7/(k_8 + k_9)$ and $c_4 = k_{10}/(k_{11} + k_{12})$.
Thus, in summary the independent variables are chosen as follows: (K_a, A, A_{pp}), where K_a is the total activated kinase as defined above. Then, using the expression of the steady of the complexes, the differential equations for these variables become (we use the same notations for the chemical variables and for their concentrations from now on):

$$\dot{K}_a = k_{a,K} S_1 K_{tot} - \left(k_{a,K} S_1 + \frac{k_{i,K}}{1 + c_1 A + c_2 A_p} \right) K_a \quad (3)$$

$$\dot{A} = k_{12} P_{tot} \frac{c_4 A_p}{1 + c_4 A_p + c_3 A_{pp}} - k_3 K_a \frac{c_1 A}{1 + c_1 A + c_2 A_p} \quad (4)$$

$$\dot{A}_{pp} = k_6 K_a \frac{c_2 A_p}{1 + c_1 A + c_2 A_p} - k_9 P_{tot} \frac{c_3 A_{pp}}{1 + c_4 A_p + c_3 A_{pp}} \quad (5)$$

where A_p is the solution of the conservation equation of A_{tot}:

$$A_{tot} = A + A_p + A_{pp} + K_a \frac{c_1 A + c_2 A_p}{1 + c_1 A + c_2 A_p} + P_{tot} \frac{c_4 A_p + c_3 A_{pp}}{1 + c_4 A_p + c_3 A_{pp}} \quad (6)$$

For convenience of notations, let us define two functions $f_1(K_a, A, A_p) = K_a \frac{c_1 A + c_2 A_p}{1 + c_1 A + c_2 A_p}$ and $f_3(A_p, A_{pp}) = P_{tot} \frac{c_4 A_p + c_3 A_{pp}}{1 + c_4 A_p + c_3 A_{pp}}$, and rewrite eq.(6) as follows

$$A_{tot} = A + A_p + A_{pp} + f_1(K_a, A, A_p) + f_3(A_p, A_{pp}) \quad (7)$$

Dimension reduction of the "1+2" model

A model of the complete MAPK cascade usually considers three levels of covalent modification cycles, where the first one is a single phosphoprylation cycle, and the two others are double phosphorylation cycles [5]. The SK model can be viewed as a signaling motif simplifying the MAPK cascade by considering a double phosphorylation cycle with the activation of the kinase. Another simplification of the MAPK cascade, but slightly more complex than the SK model, is to take into account the two first levels of the cascade. This model, which is named the "1+2" model in the main text, contains now 9 independent variables. It has been studied by Qiao et al who show the existence of sustained oscillations via a numerical study [2]. In [3] the existence of sustained oscillations in this system was studied in a reduced model to 3 equations, using the quasi-steady approximations for the intermediate complexes. We will use the same framework here. In particular the same variables can be considered, namely (K_a, A, A_{pp}) as introduced in the previous section.
Therefore, using the same method as in [3], the dynamics of the "1+2" model are ruled by the following system of equations:

$$\dot{K}_a = a_3 E_{1tot} \frac{c_0 K_0}{1 + c_0 K_0} - a_6 E_{2tot} \frac{c'_0 K_a}{1 + c_1 A + c_2 A_p + c'_0 K_a} \quad (8)$$

$$\dot{A} = k_{12} P_{tot} \frac{c_4 A_p}{1 + c_4 A_p + c_3 A_{pp}} - k_3 K_a \frac{c_1 A}{1 + c_1 A + c_2 A_p} \quad (9)$$

$$\dot{A}_{pp} = k_6 K_a \frac{c_2 A_p}{1 + c_1 A + c_2 A_p} - k_9 P_{tot} \frac{c_3 A_{pp}}{1 + c_4 A_p + c_3 A_{pp}} \quad (10)$$

where c_0 and c'_0 are the inverses of the Michaelis-Menten constants of the first covalent modification cycle. Parameters E_{2tot} is the total phosphatase for the first cycle and the "input signal" E_{1tot} represents the total input kinase of this first cycle. In the above differential equation K_0 and A_p are deduced from the independent variables K_a, A and A_{pp} thanks to conservation laws. On the one hand, A_p is the solution of the conservation equation of A_{tot} already met with eq. 7. On the other hand, K_0 is obtained by solving a new conservation of the total kinase in the first level of the "1+2" model. The latter can be written as:

$$K_{tot} = K_0 + K_a + E_{1tot}\frac{c_0 K_0}{1 + c_0 K_0} + E_{2tot}\frac{c'_0 K_a}{1 + c_1 A + c_2 A_p + c'_0 K_a} \tag{11}$$

Interaction graphs of the reduced models

A biochemical network described by a set of coupled ordinary differential equations can be associated to a so-called *interaction graph* in the following way. The nodes of the graph are defined by the biochemical species, e.g. $(x_1, \cdots, x_m)$. Then, according to the sign of $\sigma_{ij} = sign(\frac{\partial \dot{x}_i}{\partial x_j})$, the variables x_i and x_j are connected by a signed arrow going from x_j to x_i (and if $\sigma_{ij} = 0$ there is no arrow). This information can serve to characterize the instantaneous influence of the variable x_j on the variable x_i in the interaction graph. For example $\sigma_{ij} > 0$ means that the rate of change of x_i increases if a positive perturbation of x_j occurs, and conversely decreases if the perturbation of x_j is negative. Therefore a positive edge from x_j to x_i tells one that variable x_j exerts a *positive interaction* on x_i, or a *negative interaction* if the sign σ_{ij} is negative. Moreover a closed circuit along the interaction graph reveals the presence of a *feedback loop* inside the system, whose sign is the product of the interaction signs along the considered circuit. In several instances of biological networks although the value of the Jacobian matrix depends on x, its sign is constant in the phase space [6]. In this case it is meaningful to qualitatively characterize the biochemical network by its interaction graph, as it gives worthwhile information on the possible behaviors of the dynamical system. To recall only two main results, the existence of a positive feedback loop in the interaction graph is a necessary condition to confer multistability to the system [7], whereas the existence of a limit cycle requires the existence of a negative feedback loop (more precisely in the latter case the negative feedback loop is required in the non-oriented interaction graph, see [8]).

Let us apply this result to the signaling motifs obtained above. The reduced system of equations (3)-(5) modeling the signaling motif can be characterized by a 3-node network of interacting variables (K_a, A, A_{pp}). In this small network, let us calculate the sign of the interaction that A_{pp} exerts on K_a:

$$\begin{aligned}\frac{\partial \dot{K}_a}{\partial A_{pp}} &= -\frac{\partial}{\partial A_{pp}}\left[\frac{k_{i,K}}{1 + c_1 A + c_2 A_p}\right] \\ &= \frac{c_2 k_{i,K}}{(1 + c_1 A + c_2 A_p)^2}\frac{\partial A_p}{\partial A_{pp}}\end{aligned} \tag{12}$$

and $\frac{\partial A_p}{\partial A_{pp}}$ can be computed by computing the differential of eq.(7):

$$\frac{\partial A_p}{\partial A_{pp}} = -\frac{\left(1+\frac{\partial f_3}{\partial A_{pp}}\right)}{\left(1+\frac{\partial f_1}{\partial A_p}+\frac{\partial f_3}{\partial A_p}\right)} < 0 \tag{13}$$

The negative sign of the result is ensured by the positive sign of $\frac{\partial f_1}{\partial A_p}$, $\frac{\partial f_3}{\partial A_p}$ and $\frac{\partial f_3}{\partial A_{pp}}$.
Therefore one can say that variable A_{pp} exerts a *negative interaction* on variable K_a since, according to eq.(12), it always true that $\frac{\partial \dot{K}_a}{\partial A_{pp}} < 0$.
Let us now evaluate the feedback by which K_a acts on A:

$$\begin{aligned}\frac{\partial \dot{A}}{\partial K_a} &= \left(c_4 k_{12} P_{tot} \frac{1+c_3 A_{pp}}{(1+c_4 A_p + c_3 A_{pp})^2} + c_2 k_3 K_a \frac{c_1 A}{(1+c_1 A + c_2 A_p)^2}\right) \frac{\partial A_p}{\partial K_a} \\ & -k_3 \frac{c_1 A}{1+c_1 A + c_2 A_p}\end{aligned}$$

and

$$\frac{\partial A_p}{\partial K_a} = -\frac{\frac{\partial f_1}{\partial K_a}}{\left(1+\frac{\partial f_1}{\partial A_p}+\frac{\partial f_3}{\partial A_p}\right)} < 0 \tag{14}$$

which is always negative because $\frac{\partial f_1}{\partial A_p}$, $\frac{\partial f_3}{\partial A_p}$ and $\frac{\partial f_1}{\partial K_a}$ are positive.
Therefore there is a negative interaction of K_a on A.
It is also instructive to compute the mutual interactions between A and A_{pp}.
These are given respectively by:

$$\begin{aligned}\frac{\partial \dot{A}_{pp}}{\partial A} &= -k_6 K_a \frac{c_1 c_2 A_p}{(1+c_1 A + c_2 A_p)^2} \qquad (15) \\ & + \left(c_2 k_6 K_a \frac{1+c_1 A}{(1+c_1 A + c_2 A_p)^2} + k_9 P_{tot} \frac{c_3 c_4 A_{pp}}{(1+c_4 A_p + c_3 A_{pp})^2}\right) \frac{\partial A_p}{\partial A} \\ \frac{\partial \dot{A}}{\partial A_{pp}} &= -k_{12} P_{tot} \frac{c_3 c_4 A_p}{(1+c_4 A_p + c_3 A_{pp})^2} \qquad (16) \\ & + \left(c_4 k_{12} P_{tot} \frac{1+c_3 A_{pp}}{(1+c_4 A_p + c_3 A_{pp})^2} + k_3 K_a \frac{c_1 c_2 A}{(1+c_1 A + c_2 A_p)^2}\right) \frac{\partial A_p}{\partial A_{pp}}\end{aligned}$$

These two interactions are again negative because

$$\frac{\partial A_p}{\partial A} = -\frac{\left(1+\frac{\partial f_1}{\partial A}\right)}{\left(1+\frac{\partial f_1}{\partial A_p}+\frac{\partial f_3}{\partial A_p}\right)} < 0 \tag{17}$$

and $\frac{\partial A_p}{\partial A_{pp}} < 0$ following eq.(13).
On the other hand, the same type of computation shows that the interactions $A \rightarrow K_a$ and $K_a \rightarrow A_{pp}$ do not have a constant sign. They might be positive or negative. Nevertheless, from the above computations we can infer that the interaction graph of the network motif of Fig. A1 includes two feedback loops : (i) A positive feedback loop going around A and A_{pp}, which is essential for the existence of bistability in the double phosphorylation cycle between A and

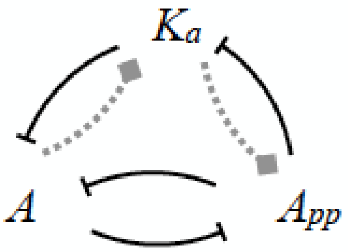

Figure A1: Network of the feedbacks between the variables K_a, A and A_{pp} of the reduced Suwanmajo and Krishnan signaling motif.

A_{pp}; (ii) A negative feedback loop in the circuit $K_a - A - A_{pp} - K_a$, which is necessary to get sustained oscillations in the SK signaling motif.
Not surprisingly, an interaction graph with the same structure can be identified in the reduced model of the "1+2" signaling motif. Both models differ only by one equation, eq. (3) of the SK model being replaced by eq. (8) in the 1+2 system. Therefore only one interaction term should be computed, namely $\frac{\partial \dot{K}_a}{\partial A_{pp}}$ which should found to be negative. This is given by:

$$\frac{\partial \dot{K}_a}{\partial A_{pp}} = k_a E_{1tot} \frac{c_0}{(1+c_0K_0)^2} \frac{\partial K_0}{\partial A_{pp}} + k_i E_{2tot} \frac{c_2 c_0' K_a}{(1+c_1A+c_2A_p+c_0'K_a)^2} \frac{\partial A_p}{\partial A_{pp}} \tag{18}$$

The factor $\frac{\partial K_0}{\partial A_{pp}}$ can be worked out by computing the differential of (11), and writes:

$$\frac{\partial K_0}{\partial A_{pp}} = E_{2tot} \frac{c_2 c_0' K_a}{(1+c_1A+c_2A_p+c_0'K_a)(1+E_{1tot}c_0/[1+c_0K_0]^2)} \frac{\partial A_p}{\partial A_{pp}} \tag{19}$$

Thus the sign of $\frac{\partial \dot{K}_a}{\partial A_{pp}}$ is that same as the one of $\frac{\partial A_p}{\partial A_{pp}} < 0$, as was already shown above.
Therefore one obtains the same network graph (Fig. A1) for both the SK signaling motif and the 2-layer truncated MAPK cascade. So, in both signaling motifs there are two interlinked feedbacks, positive and negative, whose relative strength is modulated by the biochemical parameters. As explained in the main text, parameter k_3 enhances the strength of the negative feedback loop and weakens the positive feedback loop, leading to a transition of oscillations of type 1 to type 2. In conclusion, the type of oscillations which can emerge in the network highly depends on the balance between these two feedbacks.

References

[1] Suwanmajo T, Krishnan J. Exploring the intrinsic behaviour of multisite phosphorylation systems as part of signalling pathways. *Journal of The Royal Society Interface.* 2018;15(143):20180109.

[2] Qiao L, Nachbar RB, Kevrekidis IG, Shvartsman SY. Bistability and oscillations in the Huang-Ferrell model of MAPK signaling. *PLoS Computational Biology.* 2007;3(9):e184.

[3] Sepulchre JA, Ventura AC. Intrinsic feedbacks in MAPK signaling cascades lead to bistability and oscillations. *Acta Biotheoretica.* 2013;61(1):59–78.

[4] Cornish-Bowden A. *Chapter 2 - Introduction to enzyme kinetics. In: Fundamentals of Enzyme Kinetics.* pp. 16-38. Butterworth-Heinemann. 1979.

[5] Huang CY, Ferrell JE. Ultrasensitivity in the mitogen-activated protein kinase cascade. *Proceedings of the National Academy of Sciences.* 1996; 93(19):10078–10083.

[6] Thomas R, d'Ari R. *Biological feedback.* CRC press. 1990.

[7] Soulé C. Graphic requirements for multistationarity. *ComPlexUs.* 2003; 1(3):123–133.

[8] Gouzé JL. Positive and negative circuits in dynamical systems. *Journal of Biological Systems.* 1998;6(01):11–15.
